# Recommendations for the primary prevention of atherosclerotic cardiovascular disease in primary care: a systematic guideline review

**DOI:** 10.3389/fmed.2024.1494234

**Published:** 2025-01-21

**Authors:** Maren Bredehorst, Ana I. González-González, Lara Schürmann, Dennis Firmansyah, Christiane Muth, Jörg Haasenritter, Veronika van der Wardt, Svetlana Puzhko

**Affiliations:** ^1^Department of Primary Care, University of Marburg, Marburg, Germany; ^2^Department of General Practice and Family Medicine, Medical School OWL, University Bielefeld, Bielefeld, Germany

**Keywords:** cardiovascular disease, atherosclerosis, primary prevention, adult, clinical guideline, systematic review, risk assessment, counseling

## Abstract

**Introduction:**

This study systematically reviews and synthesizes recommendations from national and international clinical practice guidelines (CPGs) regarding the primary prevention of atherosclerotic cardiovascular disease (ASCVD) in adults in primary care settings.

**Methods:**

CPGs were retrieved from MEDLINE, Trip, guideline repositories, and websites of guidelines-producing societies. Two reviewers independently screened the guidelines for eligibility, assessed their quality, and extracted study characteristics and relevant recommendations for further consistency analysis. Recommendations, with their strength and evidence level, were thematically coded and clustered around clinical questions using ATLAS.ti.

**Results:**

We included 26 CPGs from which we extracted 581 recommendations on risk assessment, non-pharmacological, and pharmacological interventions. Twenty-one guidelines (81%) were rated as having “very good” methodological quality. We categorized the recommendations into 124 clusters. Forty-four clusters (35%) included consistent recommendations, but only four of them (3%) included highly consistent recommendations. These clusters emphasized avoiding routine prescriptions of nicotinic acid, aspirin, and fibrates for primary ASCVD prevention alone, and recommending 20 mg/day of atorvastatin for high-risk ASCVD patients. The recommendations also highlighted the importance of adhering to a Mediterranean-type diet, patient-centered counseling, and standardized risk assessment for patients over the age of 40.

**Discussion:**

This review underscores the heterogeneity in primary ASCVD prevention recommendations and the importance of personalized strategies for at-risk individuals.

**Systematic review registration:**

PROSPERO, CRD42023394605, available from: https://www.crd.york.ac.uk/prospero/display_record.php?ID=CRD42023394605.

## 1 Introduction

Atherosclerotic cardiovascular diseases (ASCVDs) are a leading cause of mortality and morbidity worldwide, significantly burdening healthcare systems and economies (1). The primary prevention is crucial for reducing the incidence, consequential premature deaths and overall burden (2–7). As scientific knowledge and medical advancements continue to evolve, periodical updates of guidelines are needed to ensure the adoption of the most effective and evidence-based approaches.

Emerging evidence on factors detrimental or beneficial to cardiovascular health has shed new light on the optimal approaches for prevention. Recent studies have highlighted the importance of addressing novel risk factors, such as air pollution, sedentary lifestyle, and psychosocial stress (8, 9). While acknowledging that individual interventions provided by physicians are not always best suited to address these factors, integrating such new findings into a clinical practice guideline will enhance its comprehensiveness and relevance in addressing contemporary challenges.

Advancements in diagnostic techniques and risk assessment tools have enabled a more precise identification of individuals at high risk of developing ASCVDs. Family physicians are confronted with the question of whether tools such as coronary artery calcium scoring and genetic testing for familial hypercholesterolemia should be used in the field of primary prevention to facilitate early detection, enable timelier intervention and support more personalized preventive strategies (10).

Furthermore, the evolving landscape of pharmacotherapy for ASCVD prevention necessitates an update of guidelines. New classes of drugs, such as proprotein convertase subtilisin/kexin type 9 (PCSK9) inhibitors and sodium-glucose cotransporter-2 (SGLT2) inhibitors, may provide significant cardiovascular benefits as it has been proved in a recent meta-analysis (11, 12). Integrating evidence on novel therapies, but also novel evidence on commonly applied therapies including non-pharmacological measures, into the updated guideline may optimize treatment options for high-risk individuals and avoid harm.

Lastly, the socioeconomic and demographic characteristics of the population targeted by a clinical guideline are continuously undergoing changes. Also, the conception of subgroups regarding mechanisms of discrimination and stigmatization is changing, as can be seen in the evolving literature on gender or ethnicity and cardiovascular health (13). Considering factors such as an aging and diversifying population, increasing rates of frailty, multimorbidity and polypharmacy, and health disparities among certain subgroups, is crucial for designing effective and equitable preventive strategies.

The update of a primary prevention guideline for cardiovascular diseases in a national context is warranted to incorporate new evidence, advancements in diagnostic tools, emerging therapeutic options, and evolving population characteristics. By ensuring that the guideline reflects the latest scientific knowledge, the recommendations will support healthcare professionals in delivering optimal preventive care, reducing the burden of ASCVDs, and improving the overall cardiovascular health of the population. The objective is to conduct a systematic review of national and international guidelines to ensure the updated ASCVD prevention guidelines are evidence-based, comprehensive, and tailored to diverse populations, with special consideration for age, ethnicity, sex, gender, and specific health conditions like diabetes, multimorbidity, and polypharmacy.

## 2 Materials and methods

We conducted a systematic guideline review of clinical practice guidelines (CPGs) focused on the primary prevention of atherosclerotic cardiovascular events. Our review adhered to the systematic guideline review methodology outlined by Muth et al. (14, 15) and was registered with PROSPERO (ID CRD42023394605). The study was reported according to the Preferred Reporting System Items for Systematic Review and Meta-Analysis (16) (Supplementary Table S1). We have described the methodology in more detail in a study protocol (17).

### 2.1 Inclusion and exclusion criteria

We included CPGs that offered recommendations for the primary prevention of cardiovascular events in adults without a history of ASCVD. These guidelines needed to be evidence-based, providing references to underlying evidence, and assessing the benefits and harms of different care options (18). To focus on recent evidence since the last update of the German guideline in 2016, we considered guidelines published or updated in English, Spanish, German, or Dutch language after 2016. We excluded expired guidelines, those not authorized by professional organizations, and those not utilizing a global ASCVD risk assessment approach (19).

#### 2.1.1 Target population

The guidelines included in our review targeted individuals aged 18 and above without a diagnosis of manifest ASCVD. These guidelines adopted a holistic approach to managing overall ASCVD risk by typically addressing various risk factors prevalent in the general population, such as type 2 diabetes, hypertension, smoking, dyslipidemia, obesity, sedentary lifestyle, and unhealthy diet. Guidelines focused on specific populations (i.e., pregnant women) or healthcare processes unrelated to primary ASCVD prevention (e.g., bridging in preparing for surgery) were excluded, unless they directly addressed primary prevention of ASCVD under specific health conditions (e.g., rheumatic conditions, psychoses).

#### 2.1.2 Type of interventions

Our review encompassed guidelines covering diverse primary care interventions for preventing cardiovascular events. This included risk assessment and communication, non-pharmacological interventions, and pharmacological interventions.

#### 2.1.3 Scope of the included guidelines

We included guidelines that predominantly addressed the primary prevention of ASCVD and adopted a global risk approach.

### 2.2 Information sources

We conducted a literature search using multiple sources, including MEDLINE via PubMed and the “Turning research into practice” (Trip) database. We used Medical Subject Headings (MeSH) terms and keywords related to cardiovascular diseases, primary prevention, and clinical practice guidelines. The search was limited to publications from 2016 onwards to ensure the inclusion of the most recent and relevant evidence since the guideline to update was published in 2016. We also searched guideline-specific databases and websites of societies in cardiology, hypertension, diabetes, and primary care.

### 2.3 Selection process

We managed data for title and abstract screening and full-text assessment with the help of Zotero (20) and Covidence Systematic Review Software (21). Two independent reviewers screened titles and abstracts, with a calibration test to ensure agreement, achieving the target of 80% agreement in a sample of 30 records during text and abstract screening. Full-text assessment of potentially relevant guidelines was conducted independently by two reviewers, with disagreements resolved through discussion or involvement of a third reviewer if needed. Guideline repositories and societies’ websites were hand-searched, and references of included guidelines were checked for additional CPGs.

### 2.4 Dealing with duplicate records

We used Zotero (20) and Covidence Systematic Review Software (21) to manage and eliminate duplicate records. Our process ensured a consolidated and non-redundant collection of CPGs by integrating records from various sources.

### 2.5 Data collection and data items

Data extraction involved two stages. In the first stage, we collected guideline characteristics, as a basis for assessing their methodological quality (see section 2.6). In the second stage, we extracted individual recommendations for qualitative data analysis using ATLAS.ti software (22). Recommendations outside the scope of primary ASCVD prevention in primary care were excluded. The extraction process included identifying clinically relevant questions underlying the recommendations by orienting codes to the elements of the PICO scheme (23), extracting the recommendation content by quoting the wording, determining the grade of recommendation and level of evidence as assigned by the guideline authors, and identifying the literature cited in support of the recommendation.

ATLAS.ti (22) aided in the targeted retrieval of coded text segments and in the comprehensive analysis of the extracted recommendations. The extracted recommendations were first broadly categorized as related to risk assessment including patient-provider interaction, non-pharmacological interventions, and pharmacological interventions. These categories were further broken down into subcategories according to the intervention-related codes. Finally, the recommendations were clustered by specific clinical questions. The recommendations included in each cluster (i.e., different guidelines’ answers to the identified clinical questions) were then subjected to consistency analysis (see section 2.7). Sub-population interventions were double coded to allow further analysis.

### 2.6 Assessment of quality

Guideline quality was assessed using the MiChe list (24, 25), previously used by our research group, which involves eight specific questions and two holistic items. These dimensions include the identification of key recommendations, specification of the target audience and scope, definition of objectives and target population, independence and conflict of interest disclosure, systematic search for evidence and selection criteria, clarity of recommendations, discussion of different treatment options, and information on update procedures. A Likert scale, ranging from “1” (very good) to “7” (very poor), was used for the assessment. Any discrepancies were resolved through discussion or involvement of a third reviewer.

### 2.7 Consistency analysis

Consistency analysis compared the content (i.e., scope and direction), and level of evidence and grading assigned by the guideline authors for recommendations regarding similar key questions across different guidelines (26). We applied seven categories of consistency based on the above-mentioned factors, as this analysis is crucial for identifying gaps in the evidence and inconsistencies between guidelines, which in turn supports the development of a further action plan for guideline refinement and highlights areas where additional research is needed. The original framework was modified by adding Type C (low inconsistency) to the two types of inconsistency: Type A (high inconsistency) and Type B (moderate inconsistency). The four types of consistency—Type 1 (high consistency with a large body of high-level evidence), Type 2 (high consistency with a small body of high-level evidence), Type 3 (high consistency with low-level evidence or expert consensus), and Type 4 (high consistency with conflicting evidence)—along with a “not ratable” category for unique, non-comparable recommendations from single guidelines (Supplementary Table S2). This classification highlights that while the content of recommendations remains consistent across Types 1–4, the varying levels and reliability of evidence necessitate different approaches for guideline updates and research prioritization.

Any discrepancies that arose during the consistency assessment process were addressed through discussion and consensus, with the involvement of a third reviewer if required.

### 2.8 Data synthesis

Key findings and extracted recommendations are presented in tables and synthesized narratively. The data were structured primarily around the interventions in clinically relevant questions (i.e., clusters) derived from the pre-defined analytical framework (code system) and then refined by qualitative text analysis. Population sub-groups and corresponding interventions were also categorized and knowledge gaps for further research were highlighted. The frequency, content, and level of (in)consistency of recommendations were summarized, also disclosing the respective source guidelines, to display the “landscape” of the primary prevention of ASCVD in primary care populations.

## 3 Results

### 3.1 Literature search and selection

Following a thorough screening process of 5,123 references, we included 26 CPGs from 15 different organizations in our systematic review (Supplementary Figure S1).

### 3.2 Key characteristics of included CPGs

Out of these, 17 guidelines were developed in North America (5, 7, 26–40), and seven originated from Europe (41–47). All included guidelines were published in English, while guidelines in other languages (i.e., German, Dutch, Spanish) were also considered eligible (Table 1; Supplementary Table S3).

**Table 1:** Descriptive summary of included guidelines.

**Variable**	**Total – n (%)**
*Guidelines characteristics*	
Geographical location	
North America	17 (65.39)
Europe	7 (26.92)
South America	1 (3.85)
Asia	1 (3.85)
Scope	
Management of ASCVD risk factors in general	5 (19.23)
Management of ASCVD risk factors in rheumatic disease	1 (3.85)
Management of prediabetes / diabetes	3 (11.54)
Management of dyslipidemia	4 (15.38)
Management of weight loss	1 (3.85)
Promote healthy behaviors	4 (15.38)
Benefits and harms of using non-traditional risk factors	3 (11.54)
Benefits and harms of hormone replacement therapy	2 (7.69)
Benefits and harms of aspirin	1 (3.85)
Benefits and harms of vitamin, mineral, and multivitamin supplementation	1 (3.85)
Target audience	
Primary care professionals only	13 (50.00)
Healthcare professionals in general	12 (46.15)
Target population	
Adults 18 or older without ASCVD	5 (19.23)
Adults 18 or older without ASCVD with psychosis	1 (3.85)
Adults 18 or older without ASCVD with ASCVD risk factors	1 (3.85)
Adults without ASCVD with hypertension	1 (3.85)
Adults without ASCVD with type 1 or type 2 diabetes	1 (3.85)
Adults without ASCVD with metabolic syndrome	1 (3.85)
Adults 40 or older without ASCVD	2 (7.69)
Adults 40 or older without ASCVD with dyslipidemia	1 (3.85)
Adults 40 or older without ASCVD with dyslipidemia with high dose statins or intolerant	1 (3.85)
Adults 35–70 without ASCVD with obesity	1 (3.85)
Perimenopausal and postmenopausal women	2 (7.69)
Adults with and without ASCVD	3 (11.54)
Adults without stroke	2 (7.69)
Adults with multimorbidity	1 (3.85)
Adults with rheumatic disease	1 (3.85)
Outcomes	
ASCVD	25 (96.15)
Stroke	1 (3.85)

*ASCVD = Atherosclerotic Cardiovascular Disease.*

### 3.3 Appraisal of the methodological quality

Out of the 26 guidelines assessed for quality, 21 (81%) demonstrated a high level of quality, rated as “very good” (5, 7, 26–38, 42–46, 48). Five guidelines exhibited minor shortcomings related to systematic search reporting and selection criteria (39–41, 47, 49) (Supplementary Table S4).

### 3.4 Framework development and data extraction

Our analytical framework encompassed three domains of intervention, reflected as main categories in the code tree. A total of 581 recommendations (Median: seven recommendations per guideline; IQR: 38) were extracted across the guidelines, with pharmacological interventions (*n* = 254) being the most frequently coded, followed by non-pharmacological interventions (*n* = 224) and risk assessment including patient-provider interaction (*n* = 166). If recommendations encompassed multiple intervention types or referred to specific populations, we double coded them for our analysis. Hence, the clusters in the three domains, as presented in section 3.6, may thematically overlap if they are related to the same patient group (e.g., those with specific risk factors).

Extracted data was further sorted in terms of comparability between recommendations on similar topics covered by the different guidelines. Clusters of recommendations were formed if comparability was given regarding the underlying clinical question. As a result, 124 clusters were created: (i) risk assessment and patient-provider interaction (*n* = 24 clusters), (ii) non-pharmacological interventions (*n* = 52 clusters), and (iii) pharmacological interventions (*n* = 32 clusters). These clusters underwent a consistency analysis, whereas the remaining recommendations from single guidelines that could not be clustered were classified as “not ratable.”

### 3.5 Consistency and inconsistency of recommendations

To assess the consistency of recommendations, we examined the content and evidence grading classifications applied within the 124 clusters from the included CPGs. The analysis showed significant variability. This variability appeared in several areas, including the scope of the recommendations, the methods used to develop evidence-based recommendations versus those based on expert consensus (Supplementary Table S5), and the final direction and strength assigned by the guideline authors.

**Figure 1:**
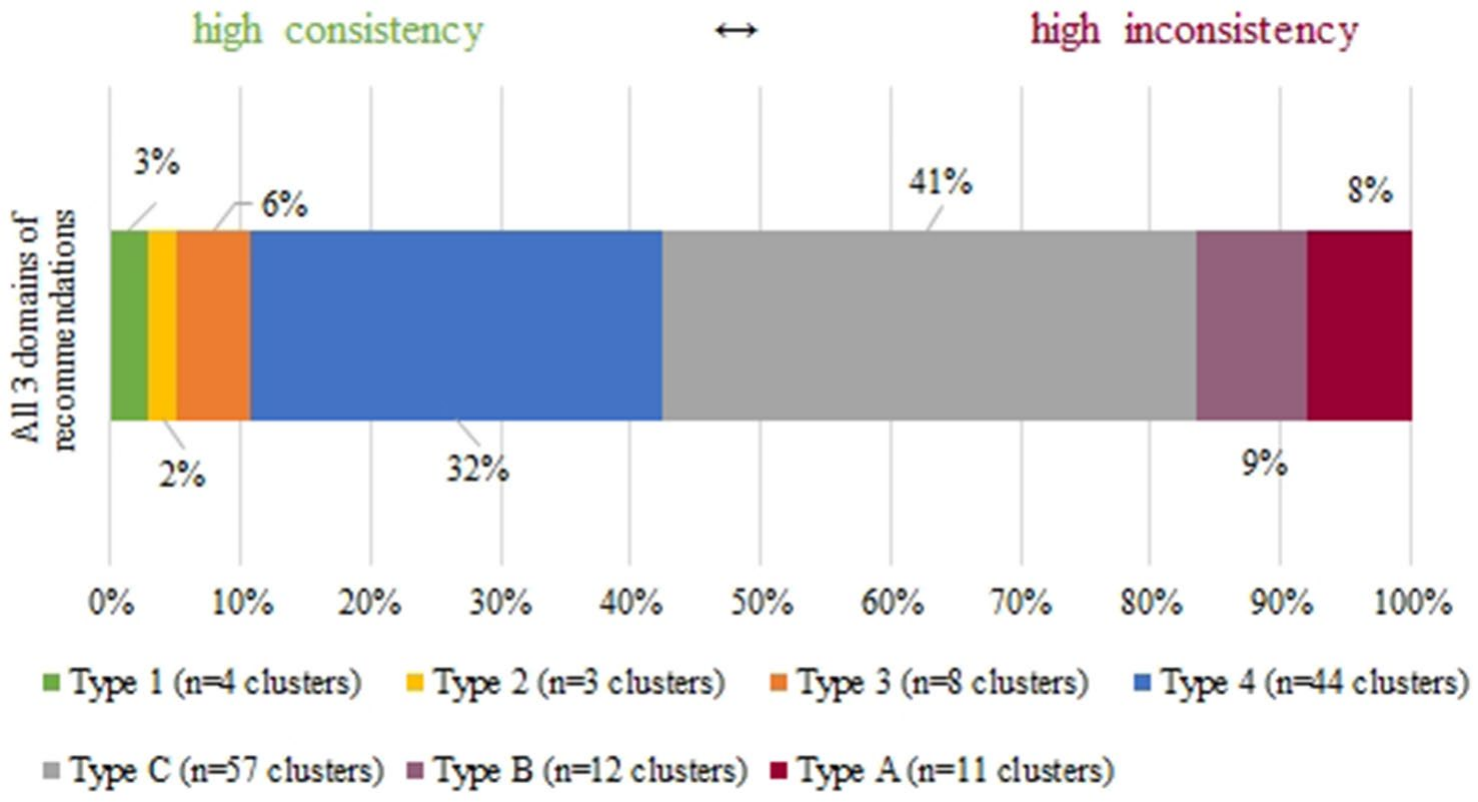
Spectrum of consistency within clusters of recommendations.

**Figure 2:**
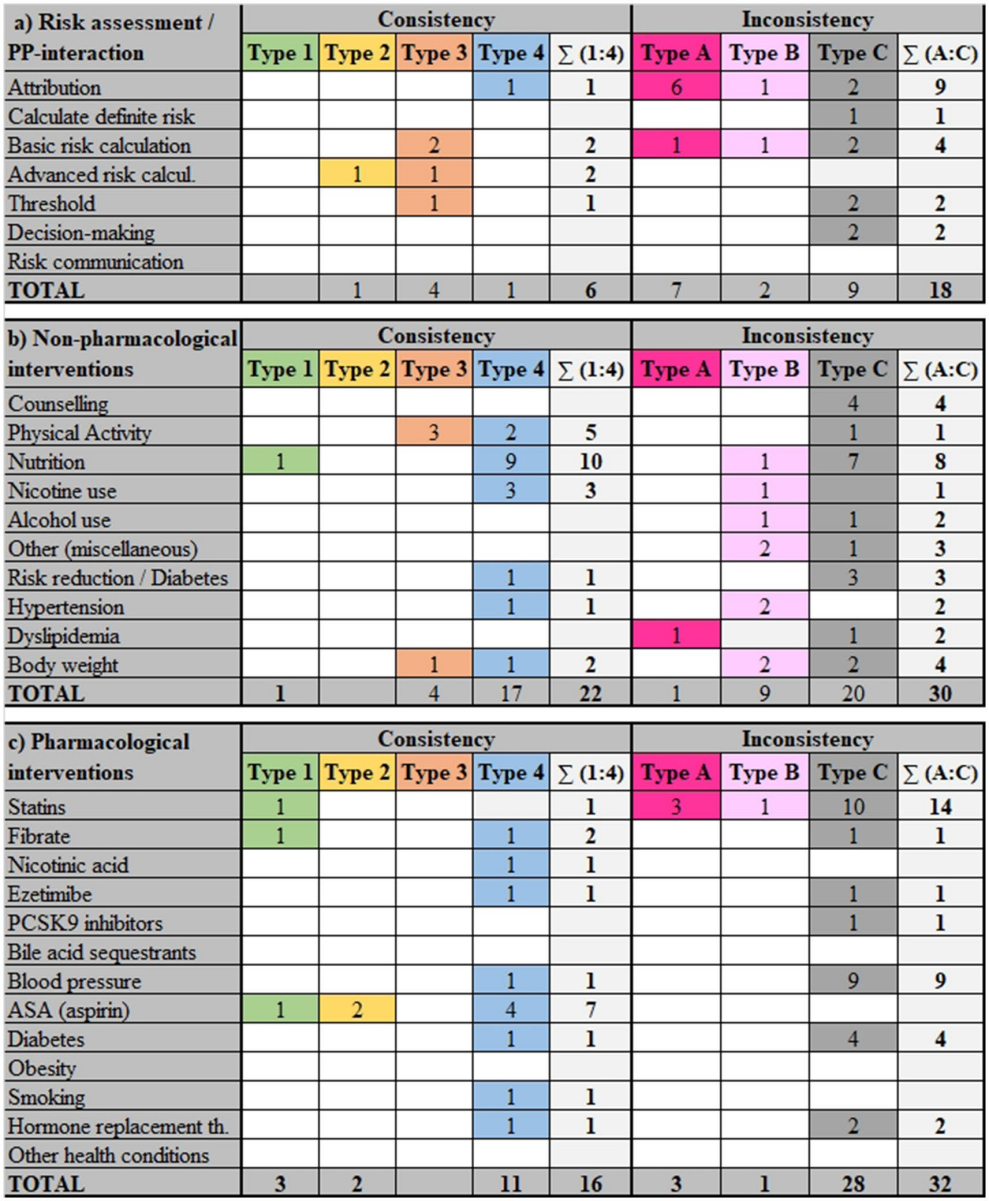
Distribution of consistency and inconsistency types across intervention domains.

Resulting from the comparison of content and direction plus the level of supporting evidence and strength of recommendation, areas of consistency and inconsistency in each domain of intervention emerged, which are further described in section 3.6. Overall, 44 clusters (35%) were consistent, and four of these (3%) showed Type 1 consistency (Figure 1). These clusters primarily focused on avoiding routine prescriptions of nicotinic acid, aspirin, and fibrates for primary ASCVD prevention alone (strong recommendations against) and recommending 20 mg/day of atorvastatin for high-risk ASCVD patients (strong recommendation for). Toward the other end of the spectrum, recommendations within 80 different clusters (65%) were inconsistent, with 11 (9%) of these including high inconsistent recommendations (Type A). The topics of these clusters also included statin indication and dosage, as well as risk reduction strategies in persons with dyslipidemia. Besides, the attribution of formal risk assessment as well as methods of risk calculation emerged as a contested field. The largest categories are those of Type C inconsistency (*n* = 57 clusters; 46%), followed by Type 4 consistency (*n* = 29; 23%). They comprise most clusters in all three domains of interventions (Figure 2). In addition, recommendations that were not ratable in our consistency analysis outnumber the clusters: 136 topics were only covered by one or more recommendations in a single guideline, which also reflects heterogeneity between guidelines.

### 3.6 Summary of international recommendations on primary prevention of cardiovascular disease guidelines

We provide in Supplementary Table S6 an overview of the clinical questions covered in the included guidelines for the primary prevention of ASCVD, outlining the various recommendations aimed at reducing its risk. The levels of consistency or inconsistency within clusters addressing the different types of intervention are also shown in Supplementary Table S6.

#### 3.6.1 Recommendations on risk assessment and patient-provider interaction

A review of 17 guidelines (5, 7, 26–28, 30, 32, 34, 37, 38, 40, 41, 43, 46, 48–50) resulted in 166 recommendations extracted for ASCVD risk assessment including patient-provider interaction. These recommendations were organized into 24 clusters, with an additional 27 recommendations that could not be included in any specific cluster. There was a total of six (25%) clusters that included consistent recommendations. However, inconsistencies were more pronounced, with 18 (75%) clusters including recommendations with varying levels of inconsistency (Figure 2). This variation suggests significant challenges in standardizing risk assessment methods, while the sphere of patient-provider interaction appears less contested.

##### 3.6.1.1 Which modalities should be considered in attributing ASCVD risk assessment?

Clinical questions concerning the attribution of formal risk assessment in primary care populations covered aspects such as age groups, clinical conditions, opportunities for and frequency of assessment.

Guidelines generally agreed on the necessity of ASCVD risk assessment for individuals aged over 40 years (7, 40, 43, 46, 50, 51). Additionally, there was variability in recommendations for younger age groups, suggesting assessments for those aged 20–39 by some guidelines and over 30 by others.

Recommendations for screening specific risk factors or conditions such as atherosclerotic plaque (43, 51), diabetes (27, 34, 40, 52), hyper−/dyslipidemia (26, 40), hypertension (32), obesity (7, 46, 50, 51), smoking/tobacco use (7, 41), and arterial stiffness (51) showed high inconsistency. There was also inconsistency across four guidelines on the use of non-invasive tests, like ECG, stress tests, and coronary computed tomography angiography for coronary artery disease screening (26, 33, 49, 51).

Three guidelines offered highly inconsistent recommendations on the use of systematic versus opportunistic screening strategies for ASCVD risk: One guideline advocated for a systematic approach (50), another recommended an opportunistic approach (48), and a third was against systematic strategies (46).

Guidelines were inconsistent on the recommended frequency of ASCVD risk assessment, suggesting various schedules and situations from annual assessments (46), to reassessments only if treatment was declined (50).

##### 3.6.1.2 What exceptions might be made from calculating the definitive ASCVD risk?

Five guidelines (7, 43, 46, 50, 52) identified groups in which a high cardiovascular risk should be assumed regardless of other risk factors or the results of a risk assessment tool. These groups primarily included individuals with diabetes, kidney disease and familial hypercholesterolemia, and other less prevalent medical conditions.

##### 3.6.1.3 Which basic ASCVD risk calculation tools are recommended?

Although there was consensus among guidelines (7, 26, 40, 41, 50, 51) in generally supporting the use of standardized risk assessment tools (e.g., QRISK2), there was significant variation in recommendations regarding which tool to use.

##### 3.6.1.4 What are the recommendations on possible advancements of ASCVD risk calculation?

Recommendations varied widely regarding the inclusion of non-traditional risk factors like coronary artery calcium (26, 30, 48, 49), ankle-brachial index (26, 30), high-sensitivity C-reactive protein (hsCRP) (26, 30), body mass index (BMI) (50) and psychosocial factors (46, 50).

On the clinical question of how cholesterol should be measured as part of an ASCVD risk assessment, there was low consistency (40, 41, 43, 46, 50) in recommendations on using measures such as total cholesterol, HDL cholesterol, triglycerides, and non-HDL cholesterol.

##### 3.6.1.5 Which thresholds for treatment are recommended for observation in ASCVD risk assessment?

Recommendations on ASCVD risk thresholds for treatment (5, 7, 26, 40, 42, 46, 49, 51, 52) were diverse and generally showed low consistency, addressing both pharmacological and non-pharmacological interventions, as well as lipid and blood pressure management and antithrombotic therapy.

##### 3.6.1.6 What recommendations exist for decision-making within the scope of managing elevated ASCVD risk?

There were moderately consistent recommendations (26, 37, 40, 46, 50, 52) for a shared decision-making approach, with one guideline endorsing it widely (7) while others (26, 37, 46, 50, 52) specifying its use for particular treatment decisions or risk levels.

Guidelines (38, 50) moderately supported informing patients about their absolute ASCVD risk as a basis for treatment decisions rather than using relative risks, although the strength of these recommendations varied.

##### 3.6.1.7 How should ASCVD risk be communicated?

There were partially consistent recommendations on risk communication methods (40, 50), with variation in language simplicity, risk personalization, and the use of visual aids.

#### 3.6.2 Recommendations on non-pharmacological interventions

We found 224 recommendations from 16 guidelines addressing non-pharmacological interventions for primary prevention of ASCVD (7, 26, 27, 29, 31, 35, 38, 40, 41, 43–49). The recommendations were categorized into 52 clusters, with an additional 34 recommendations that could not be clustered. There were 22 (42%) consistent clusters. In contrast, there were also 30 (58%) inconsistent clusters. Details can be found in Supplementary Table S6. The main issues were recommendations on lifestyle, such as physical activity and dietary measures, often combined with each other and usually embedded in behavioral counseling interventions. In this domain, recommendations for persons with specific ASCVD risk factors are therefore analyzed in the section on risk reduction (see section 3.6.2.7), rather than as sub-groups under the different intervention types.

##### 3.6.2.1 What recommendations exist for behavioral counseling in ASCVD primary prevention?

Seven guidelines (7, 26, 38, 41, 43, 45, 46) covered behavioral counseling for primary ASCVD prevention, with low inconsistency dominating. Respective recommendations included motivating and supporting patients to change risky behaviors (such as unhealthy diet and sedentary lifestyle) and provided specific counseling techniques and emphases. One guideline (31) suggested that adults aged 18 and older at elevated ASCVD risk should undergo behavioral counseling. Additionally, several guidelines offered advice on effectively delivering lifestyle and behavioral counseling. These guidelines recommended, emphasizing patient-specific circumstances and social determinants (7, 45), using positive motivational approaches (43, 45), providing written information (43, 45), and focusing on various aspects of risk communication and counseling (38, 41, 45). Training in psychological techniques (46) and a team-based approach involving referrals to dietitians were also recommended (7, 26).

##### 3.6.2.2 What are the recommendations on physical activity in preventing ASCVD?

The recommendations on physical activity can be considered as answering two types of clinical questions. On the one hand, the principal usefulness for ASCVD prevention and the possibilities of becoming more physically active are highlighted, mostly with low consistency. On the other hand, effective dosages and modalities of physical activity are addressed with partially consistent recommendations.

Nineteen recommendations addressed the issue in the sense of “activating” patients at increased risk of ASCVD. This included advice to increase activity levels or reduce sedentary behavior to lower overall ASCVD risk, specifying the type of physical activities recommended (such as cardiovascular strain, muscle strengthening, flexibility and coordination), as well as the intensity (moderate/vigorous) and frequency of these activities. Key principles for advising on physical activity included reducing sedentary behavior, encouraging routine counseling during healthcare visits (7, 46), and ensuring safe exercise levels (i.e., assessing the person’s current health, fitness level, and any medical conditions they might have to avoid injury or health complications) for encouraging moderate or more intense physical activities (45, 46).

We identified six guidelines offering recommendations on the beneficial modes of physical activity. Two guidelines (7, 26) recommended that any amount of physical activity is beneficial. Promotion of brisk walking for increased physical activity was recommended by three guidelines (27, 46, 48), and muscle-strengthening activities at least 2 days per week were endorsed by another guideline (45).

Furthermore, four guidelines provided recommendations on the beneficial levels of physical activity. Three (7, 45, 47) of these guidelines encouraged moderate physical activity for at least 150 min per week or vigorous activity for at least 75 min per week. Additionally, one guideline (40) recommended integrating physical activity into a comprehensive behavior change program.

##### 3.6.2.3 What are the nutritional recommendations for mitigating ASCVD risk?

Profound differences can be observed in the approaches of guideline authors to nutritional advice for the primary prevention of ASCVD. The scope addressed in their clinical questions reaches from broad dietary patterns, through recommendations on different types of food, to specific advice on classes of macro and micro nutrients.

Four guidelines (40, 44, 46, 51) varied significantly in their endorsement of healthy dietary patterns. Strong recommendations included the adoption of the Mediterranean diet and increased intake of fruits and vegetables (7, 44, 46, 47). Emphasis was also placed on diets focusing on plant over animal-based foods (44). Additional recommendations addressed reducing sodium, refined carbohydrates, and saturated fats by avoiding processed foods (7, 40, 44–46). One recommendation strongly supported limiting added sugar intake to less than 10% of total energy, with benefits observed when sugar intake is below 5% (48).

In terms of foods, recommendations consistently endorsed daily consumption of whole grains (44, 45, 48), at least five portions of fruits and vegetables (44, 45, 48), and moderate fish intake (44, 46, 48), though recommendations were supported by different levels of evidence. Additionally, recommendations supported advised limits on meat consumption to 1–2 times weekly and occasional processed meat (44) and daily intake of extra virgin olive oil and unsalted nuts (44, 46, 48). Recommendations also supported moderate consumption of coffee, tea, and dark chocolate (44), and advocated replacing sweetened beverages with water (44, 48).

Regarding total and saturated fat intake, recommendations suggested that total fats should comprise about 20–25% of total calorie intake with an upper safe limit of 30% (45, 48). Guidelines also advised specific daily limits for saturated fat intake based on gender and recommended keeping saturated fatty acid intake below 10% of total calories and trans fatty acids below 1% (46). Consistent advice included avoiding fat supplementation (icosapent ethyl) for primary prevention of ASCVD (26, 44–46, 48).

Guidelines consistently advocated for a reduction in sodium intake to 2 g/day (equivalent to 5 g of salt), with one guideline suggesting maintaining salt intake below 6 g/day (44, 46, 48). Recommendations also included a moderate reduction in sodium intake to decrease ASCVD risk by around 30% (48).

Two strong recommendations supported increasing dietary fiber intake to 20–30 g/day through adequate consumption of plant foods (44, 48). Recommendations on plant extracts were inconsistent; one guideline (26) found insufficient evidence to recommend fiber, garlic, ginger, green tea, and red yeast rice supplements for reducing ASCVD risks, while another advised against the use of plant stanols or sterols (45).

Overall, these dietary recommendations showed both consistency and inconsistency, often addressing different aspects or levels of detail within nutritional advice. Guidelines uniformly discouraged the use of nicotinic acid (26, 45, 46) and antioxidant vitamin supplements (35, 44, 46) for ASCVD risk reduction.

##### 3.6.2.4 What is recommended regarding nicotine use and smoking cessation to prevent ASCVD?

Six guidelines (7, 40, 45–48) provided strong recommendations, urging healthcare providers to clearly advise patients to stop smoking. The recommendations for referral to smoking cessation services suggested various support methods including pharmacotherapy, behavioral counseling, and involving dedicated professionals. These recommendations exhibit low inconsistency (7, 40, 41, 45, 48). Specific approaches for smoking cessation in individuals with mental disorders involve optimized antipsychotic treatment and tailored programs (41). Regarding pharmacotherapy for smoking cessation, moderate inconsistency is noted, with two guidelines endorsing various approaches including varenicline, combination nicotine replacement therapy, or bupropion under certain conditions (45, 46). Additional guidelines stress the importance of avoiding passive smoke exposure and prioritizing cessation efforts for young people and individuals with low incomes (7, 46–48).

##### 3.6.2.5 What are the recommendations for managing alcohol consumption in ASCVD prevention?

One guideline (46) opposed universal screening for alcohol use as a primary prevention strategy, advocating for more targeted approaches. Guidelines varied on alcohol consumption advice, including what constitutes a drink and the ASCVD risks of even moderate consumption (40, 45, 46). Recommendations for addressing hazardous drinking levels included brief, multi-contact interventions, showing moderate inconsistency in setting daily alcohol intake thresholds (one recommendation setting the limit at >30 g per day, while the other three set it at >10 g for women and > 20 g for men) (40, 41, 44, 47, 48). One recommendation (46) advised against universal screening for excess alcohol use as a case-finding exercise in primary care.

##### 3.6.2.6 What other lifestyle factors were considered in recommendations for ASCVD prevention?

Recommendations emphasized avoiding chronic exposure to stress (47) and highlighted the positive impact of mind–body practices on cardiovascular health (48). There was moderate inconsistency in recommendations for various forms of therapy, such as cognitive-behavioral therapy for depression or anxiety and various mental health services (41, 46, 48). There was also advice against the use of alternative treatments like herbal medicine, chelation therapy, ozone therapy, stem cells, and acupuncture for blood pressure management (48).

##### 3.6.2.7 What specific recommendations exist for reducing ASCVD risk in prominent risk groups?

Non-pharmacological risk reduction strategies may be addressing persons at increased cardiovascular risk in general, regardless of their specific risk factors. For example, one guideline advocated for lifestyle modification involving exercise, improved nutrition, smoking cessation, and weight control to reduce ASCVD risk (48). Other guidelines dedicated recommendations to particular risk groups such as persons with diabetes, hypertension, dyslipidemia or excess body weight, often also putting non-pharmacological measures in relation to pharmacological options, and emphasizing the consideration of co-existing risk factors.

Recommendations concerning persons with type 2 diabetes mellitus varied on the necessity of pharmacotherapy depending on individual risk levels and specific population needs (27, 40, 41, 48). There was an emphasis on managing glycated hemoglobin and blood pressure, dietary control, and smoking cessation as part of a holistic approach to managing ASCVD risk (7, 27, 40, 41, 48, 49).

In the management of hypertension, guidelines consistently advocated for reduced sodium intake (7, 40, 46). They varied in their pharmacotherapy approach, suggesting lifestyle modifications as the first-line treatment and pharmacological options only when necessary (27, 46). Recommendations for specific BP targets showed low inconsistency, with some guidelines suggesting more aggressive targets for individuals with certain risk factors (i.e., BP target if uncomplicated hypertension should be less than 140/90; if chronic kidney disease BP target should be less than 130/80 mmHg) (7, 46).

For persons with dyslipidemia, guidelines recommended lifestyle measures to reduce cholesterol levels and provided specific advice on treatment targets and medication for individuals with diabetes and high ASCVD risk (i.e., LDL-C levels reduce by 50% or more if high ASCVD risk) (46, 49). There was moderate inconsistency in recommendations regarding the monitoring of lipid levels in patients on statins (7, 26).

Guidelines consistently advocated for weight control or reduction to decrease ASCVD risk (27, 29, 40, 41, 48). They provided specific weight loss targets (i.e., 5–10% weight loss), and emphasized the use of dietary intervention combined with physical activity (40, 48). Guidelines also suggested comprehensive behavioral interventions (27, 29), particularly for obesity, and consider the role of physical activity in weight management strategies (41, 48).

#### 3.6.3 Recommendations on pharmacological interventions

Across 15 guidelines (5, 7, 26, 27, 36, 37, 40–43, 45, 46, 48, 49), we analyzed 254 recommendations classified into 48 clusters regarding pharmacological interventions for primary prevention of ASCVD. Additional 75 recommendations could not be clustered. In this domain, 16 (18%) clusters were consistent. At the same time, this domain also faced significant challenges, with 32 (67%) clusters showing discrepancies (Supplementary Table S6).

##### 3.6.3.1 What are the recommendations for the use of statins in the primary prevention of ASCVD?

Ninety recommendations from 11 guidelines (5, 7, 26, 27, 40, 42, 43, 45, 46, 49, 51) provided details on the use of statins.

Two guidelines (45, 46) offered low consistency recommendations on initiating statins for primary prevention of ASCVD, emphasizing the consideration of overall risk, potential benefits from lifestyle modifications, and clinical assessments before treatment. For individuals aged 75 and older, recommendations showed high inconsistency between two guidelines: One (27) suggested considering statin therapy in those without diabetes and a with a 10-year ASCVD risk of 7.5% or more. The other (5) cited insufficient evidence to evaluate the benefits and harms of statins for this age group. Another issue of high inconsistency was the general dosage of statins for primary prevention. Three guidelines (7, 40, 42) focused on individuals with a 10-year ASCVD risk between 7.5 and 20%, recommending initiating or intensifying statin therapy based on additional risk-enhancing factors or coronary artery calcium scores, showing low inconsistency in their approaches. Two guidelines (42, 45) addressed high ASCVD risk populations (15–20% and > 20% over 5 years), recommending with low inconsistency the consideration of additional lipid-lowering agents like ezetimibe and PCSK9 inhibitors based on individual risk levels. These two guidelines also emphasized the importance of specialist consultation in complex cases. Recommendations from two guidelines (46, 49), characterized by low inconsistency, advised initiating statin therapy in patients with chronic kidney disease (CKD) stage 3 or higher, unless contraindications such as dialysis or chronic liver disease were present. Additionally, two guidelines (46, 49) recommended statins as the primary treatment for diabetic patients with mixed dyslipidemia, occasionally in combination with fibrates if specific lipid thresholds were exceeded, with low inconsistency.

Two guidelines (45, 46) cautioned against certain drug combinations, such as the coadministration of statins with fibrates like gemfibrozil, especially in patients with risk factors for adverse interactions, with low inconsistency.

By one guideline, high-intensity statin therapy was recommended for individuals aged 40 to 75 years with an LDL-C ≥ 190 mg/dL (≥5.9 mmol/L) to achieve a reduction of ≥50% (27). However, another guideline (5) suggested a lower to moderate dose statin for adults aged 40–75 years without a history of ASCVD but with one or more ASCVD risk factors and a calculated 10-year CVD event risk of 10% or greater. This guideline cited a lack of evidence for added cardiovascular benefits from higher doses of statin. For patients with diabetes and multiple ASCVD risk factors, high-intensity statin therapy aimed at achieving an LDL-C reduction of 50% or more was advised by one guideline (7). In cases of diabetes with very high ASCVD risk, initiating statins at the highest tolerable dose was recommended by another guideline (27). Two guidelines (45, 46) recommended with high consistency offering Atorvastatin 20 mg for the primary prevention of ASCVD to high-risk patients, including those with diabetes, after a thorough risk assessment and informed discussion with their clinician. This dose was also recommended for primary prevention in patients with CKD by one guideline (45). Another guideline recommended against the use of Simvastatin 80 for primary prevention of ASCVD by one guideline (46) due to the risk of myopathy, while advising patients currently stable on this regimen to continue it for at least 1 year. Patients on medications that interact with cytochrome P450 metabolism should avoid using Atorvastatin or Simvastatin (46). Alternatives such as pravastatin or rosuvastatin were recommended instead (46).

##### 3.6.3.2 What are the recommendations for the use of non-statins in the primary prevention of ASCVD?

Thirty recommendations, classified into seven clusters, provided guidance on non-statin interventions for ASCVD prevention. These included fibrates, nicotinic acids, ezetimibe, PCSK9-inhibitors and bile acid sequestrants.

Two guidelines (45, 46) strongly advised against routinely using fibrates for primary ASCVD prevention, indicating high consistency in their recommendations. Additionally, three guidelines (26, 45, 46) discouraged the use of fibrates alongside statins, although combined therapy might be considered in cases of mixed dyslipidemia, with lower consistency noted across recommendations. Two guidelines (45, 46) recommended against using niacin for ASCVD risk reduction. Additionally, they discouraged its use in combination with statins, although with low consistency. Adding ezetimibe to statins might offer additional benefits, particularly for high-risk patients with persistently elevated cholesterol levels, according to five guidelines (26, 42, 45, 46, 49). However, evidence supporting its use for primary prevention is insufficient, showing low inconsistency. The use of PCSK9 inhibitors is suggested for high-risk patients with uncontrolled cholesterol levels despite other therapy. However, long-term safety and cost concerns persist, with low inconsistency across four guidelines (26, 42, 46, 49). Bile acid sequestrants were generally not recommended as primary therapy or in combination with statins for ASCVD prevention due to limited evidence of effectiveness, as indicated by one guideline (45).

##### 3.6.3.3 What are the recommendations for the use of blood pressure-lowering medication for the primary prevention of ASCVD?

Forty-five recommendations from six guidelines (7, 17, 31, 34, 37, 40) provided detailed guidance through the initiation and management of blood pressure (BP)-lowering medications for primary ASCVD prevention, focusing on individual risk assessment and BP thresholds. Five guidelines (7, 17, 31, 34, 37) advocated for starting BP-lowering medication in individuals with a systolic BP (SBP) of 130 mm Hg (27, 40) or 140 mm Hg (7, 46) and higher, particularly when accompanied by an ASCVD risk of 10% or more. These recommendations were characterized by low inconsistency, emphasizing personalization of treatment based on initial BP measurements and overall cardiovascular risk.

Regarding the initial choice of medication for hypertension management, two guidelines (31, 40) offered varying strengths of recommendations. Options such as diuretics, angiotensin-converting enzyme (ACE) inhibitors, angiotensin receptor blockers (ARBs), or calcium channel blockers (CCBs), were proposed, considering factors such as efficacy, tolerability, cost, and patient comorbidities, also noted with low inconsistency.

For cases where monotherapy is insufficient in achieving target BP levels, two guidelines (31, 40) provided six recommendations for combining BP-lowering medications. Recommended pairings included an ACE inhibitor or ARB with dihydropyridine CCB, explicitly advising against the concurrent use of an ACE inhibitor with an ARB. These recommendations were of low inconsistency.

Target BP levels were advised to vary based on the individual’s ASCVD risk and existing comorbidities. For patients with uncomplicated hypertension and lower ASCVD risk, targets ranged from 130/80 mm Hg to less than 140/90 mm Hg. In contrast, more stringent targets of less than 130/80 mm Hg or even ≤120 mm Hg were suggested for those at high risk of ASCVD. These recommendations also exhibited low inconsistency. For diabetic patients, aiming for BP levels below 130/80 mm Hg was recommended, though these were marked by low consistency across different guidelines (7, 31, 37, 40).

##### 3.6.3.4 What are the recommendations for the regular use of acetylsalicylic acid for the primary prevention of ASCVD?

A total of 23 recommendations across six guidelines (7, 28, 37, 40–42) were identified regarding the use of acetylsalicylic acid for primary prevention of ASCVD. Three guidelines (37, 41, 42) strongly advised against using aspirin for primary ASCVD prevention with high consistency across them; the other three guidelines advised in favor of individualizing decisions depending on the age and the estimated ASCVD risk of the person.

##### 3.6.3.5 What are the recommendations for pharmacotherapy for the treatment of diabetes mellitus?

We examined 29 recommendations from six guidelines (7, 27, 40, 41, 49) analyzing the benefits and harms of pharmacotherapy for diabetes management in the context of primary ASCVD prevention.

Three guidelines (7, 41, 49) endorsed with low inconsistency the initiation of metformin as monotherapy to enhance glycemic control and reduce ASCVD risk. Two guidelines (7, 49) suggested initiating metformin as the first-line therapy along with lifestyle changes upon diabetes diagnosis to enhance glycemic control and reduce ASCVD risk. Another guideline (41) recommended considering metformin prescription for patients not responding to intensive lifestyle interventions.

In addition to monotherapy, recommendations (7, 40, 49) for combining metformin with a second drug varied in their approach and evidence strength, characterized by low inconsistency. These recommendations varied in strength and evidence. One guideline (40) suggested sodium-glucose cotransporter 2 (SGLT-2) inhibitors or glucagon-like peptide-1 receptor (GLP-1R) agonists for patients needing additional glucose-lowering therapies despite the use of metformin and lifestyle changes. Conversely, another guideline (49) recommended the addition of a second antihyperglycemic agent if metformin and lifestyle adjustments failed to achieve target HbA1c levels above 8.5%. Furthermore, for enhanced glycemic control with a lower risk of hypoglycemia, options like incretin agents (DPP4 inhibitors or GLP1-RAs), SGLT2 inhibitors, acarbose, or pioglitazone were suggested as add-on therapies (41). The choice of pharmacotherapy was emphasized to be individualized based on efficacy, underlying mechanisms, comorbid conditions, and cost considerations.

Four recommendations from two guidelines (40, 49) related to the use of insulin in primary ASCVD prevention demonstrated low consistency due to the grade and level of evidence of the literature supporting. Insulin was positioned as a viable option for those who could not meet glycemic targets with non-insulin antidiabetic medications. Basal insulin was particularly recommended for patients unable to achieve control with existing non-insulin therapies.

The guidelines (40, 49) also specified glycemic control targets, proposing an HbA1c level below 7.0% for most adults with diabetes, with adjustments made for factors like cognitive impairment or life expectancy. For patients with type 2 diabetes and chronic kidney disease, a stricter target of ≤6.5% was recommended (41).

##### 3.6.3.6 What pharmacological interventions are recommended for other conditions in the scope of ASCVD prevention?

There are additional conditions or health behaviors relevant in the etiology of ASCVD that may be targeted by pharmacological treatment, such as obesity, tobacco smoking or postmenopause.

We analyzed 17 recommendations from three guidelines (31, 32, 42) on pharmacological interventions for weight loss in primary ASCVD prevention. One guideline supported the use of pharmacotherapy for individuals with BMI ≥ 30 kg/m2 or BMI ≥ 27 kg/m2 with related complications, alongside medical nutrition therapy, physical activity, and psychological interventions (40). Additionally, another guideline strongly recommended metformin to mitigate antipsychotic-induced weight gain, and suggested Orlistat and Glucagon-like peptide-1 (GLP-1) as effective options for weight loss when non-pharmacological methods are insufficient (49). Five guidelines (31, 32, 36, 37, 42) provided eight recommendations supporting pharmacotherapy for smoking cessation in primary ASCVD prevention. These guidelines endorsed the use of varenicline, bupropion, and nicotine replacement therapy within personalized, comprehensive smoking cessation programs that include behavioral support. However, the consistency and evidence for these recommendations were noted as low. Three guidelines (26, 30, 42) provided 16 recommendations concerning hormone replacement therapy (HRT) for primary ASCVD prevention, primarily targeting postmenopausal women. These guidelines did not recommend HRT for primary ASCVD prevention, reflecting low consistent recommendations due the different levels of the evidence supporting them.

### 3.7 ASCVD risk management in demographic and clinical sub-populations

Besides interventions aimed at ASCVD prevention, our code tree incorporated specific sub-populations encompassing demographic aspects such as age and ethnic groups, and sex/gender, as well as health conditions such as diabetes, multimorbidity, and polypharmacy (Supplementary Table S7). Where applicable, recommendations in the included guidelines were double coded with intervention codes on the one hand, and respective (sub-)population codes on the other hand. This allows for comparison with the findings presented above, to indicate approaches toward a more personalized ASCVD risk management.

A total of 17 recommendations (5, 7, 26, 27, 36, 37, 40, 45, 46, 49) centered on patient age, with a focus on the elderly (60 years and older), and some applicable to middle-aged or elderly patients (40 years and older). These recommendations covered risk assessments, medication dosages, and non-pharmacological measures.

Regarding patient sex, our review identified 24 recommendations (27, 36, 39, 41, 46, 48), with the majority concerning pharmacological interventions such as HRT in menopausal women and testosterone replacement therapy in men. A single recommendation focused on non-pharmacological measures pertaining to alcohol consumption limits (41). No specific recommendations related to patient gender were found.

There were four recommendations (27, 41, 45, 46) concerning patient ethnicity. These recommendations highlighted the importance of recording ethnicity, considering it as an ASCVD risk factor, and adjusting treatment based on ethnicity [i.e., Angiotensin-Converting Enzyme are not recommended as first-line therapy for uncomplicated hypertension in Black patients (40)].

In the context of diabetes, our review yielded 94 recommendations across guidelines (7, 27, 40–42, 45, 46, 48, 49), breaking down into 68 recommendations for pharmacological treatments, 17 for non-pharmacological interventions, and 16 for risk assessment. These recommendations encompassed various aspects of diabetes management, including medication adjustments, lifestyle modifications, and integration into comprehensive ASCVD risk evaluations.

Furthermore, 86 recommendations pertained to a spectrum of diseases and conditions with notable mentions of psychological conditions, chronic kidney disease (CKD), metabolic risks, and familial hypercholesterolemia (7, 27, 33, 40, 41, 43, 45, 46, 48, 49). These encompassed both pharmacological and non-pharmacological interventions, along with risk assessments.

Our findings included six targeted recommendations from two guidelines that addressed multimorbidity (45, 46), underlining the necessity to consider multiple concurrent conditions in treatment planning and risk assessments, thereby facilitating personalized patient care strategies.

Lastly, a total of 42 recommendations (26, 27, 41–43, 45, 46, 49) pointed to polypharmacy, primarily directing pharmacological measures, complemented by risk assessments, and a minor focus on non-pharmacological strategies. These guidelines aimed at optimizing safe and effective medication management, particularly emphasizing lipid and blood pressure control.

The recommendations across specific sub-populations or health conditions reflect the respective guideline’s emphasis on ASCVD risk, exemplified by the BAP 2016 guidelines (41) which pointed out mental illness, the SBD/SBC/SBEM 2017 (49) and CCH 2022 (40) guidelines which focused on diabetes, and the SOGC 2021 guidelines (39) which dealt with menopause and cardiovascular diseases.

## 4 Discussion

Our guideline review for the primary prevention of ASCVD has revealed a significant diversity in the recommendations provided across various guidelines. This diversity not only illustrates the complexities inherent in guideline development but also emphasizes the challenges in achieving consensus among healthcare professionals regarding the most effective strategies for ASCVD prevention. The variations observed in our review may partly be explained by the scope and focus of the individual guidelines, and subsequently the delimitation of clinical key questions to be answered, which resulted in large numbers of non-ratable recommendations. Some variations between guidelines are also indicative of different interpretations of the existing evidence, influenced by the continual evolution of new research and varied regional clinical practices, while others point to larger research gaps.

Comparing the three domains of intervention with each other, it was necessary to take differential approaches in the consistency analysis. The moderate level of agreement in the non-pharmacological domain, reflected in large amounts of clusters with low inconsistency and low consistency, is rooted in the heterogeneity of clinical questions (in particular, definitions of interventions and populations). Our adaptation of the original methodology for the consistency analysis, i.e., adding Type C as a category, was a consequence of this heterogeneity. For major pharmacological interventions, however, research questions, study designs (RCTs), and the resulting high-level evidence were expected to be more comparable than in non-pharmacological prevention studies. This expectation led us to define narrower clusters as analytical units, resulting in a greater number of clusters being classified as either highly consistent or highly inconsistent. A similarly narrow focus was applied in the sub-domain of risk assessment, whereas the sub-domain of patient-provider interaction also had a moderate level of agreement. Some divergence between recommendations on risk assessment, pharmacological and non-pharmacological interventions for particular risk groups, e.g., persons with dyslipidemia, may thus be a methodological residual in our review. Another example is the recommendation regarding vitamin B3 (niacin) use, which varies depending on whether the focus is on general dietary intake or on its use as a supplement, like medication, for specific high-risk groups.

The novelty of interventions in the primary prevention of ASCVD plays a major role in explaining variability, as do the scientific challenges of investigating the effectiveness of certain interventions and producing high-level evidence. For instance, while some interventions like statin use for certain populations showed high agreement across guidelines, newer pharmacological treatments and measures addressing risk factors such as psychosocial stress were much less frequently addressed and reached less agreement. The review therefore highlighted several critical areas that need further research. These include more research on the impact of non-traditional risk factors on ASCVD and long-term studies on the efficacy and effectiveness of complex non-pharmacological interventions and newer pharmacological agents. The development of comprehensive, personalized prevention strategies that consider individual patient profiles (health conditions and demographics, but also social, cultural and economic factors) generally requires greater attention. Further original research is essential to enhance our understanding of customizing preventive strategies based on patients’ unique characteristics, including age, gender, ethnicity, and health status.

The implications of our findings for clinical practice are profound, as inconsistency can lead to confusion and potentially impact the effectiveness of implemented prevention strategies. There is a clear need for guidelines to be adaptable and flexible, allowing for the rapid incorporation of new evidence. This adaptability starts from identifying the most relevant clinical questions for a given primary care population, and it is crucial for providing healthcare providers with the most current information to facilitate informed shared decision-making with patients. Additionally, efforts to standardize the approach to evidence grading and the formulation of recommendations could help reduce confusion and enhance the effectiveness of ASCVD prevention strategies globally.

Besides, the heterogeneity observed across recommendations may pose challenges for clinical workflows and patient outcomes. Inconsistent guidance on interventions such as statin therapy or ASCVD risk thresholds can lead to variability in practice, potentially affecting patient care quality and outcomes. To address this, healthcare systems may benefit from creating summary documents or decision aids that align with local practices and integrate social determinants of health specific to the local population while reconciling guideline discrepancies. Future research should focus on evaluating the impact of such heterogeneity on clinician adherence and patient satisfaction.

Cardiovascular health requires a tailored approach to risk assessment and management, particularly as patients reach the critical age group of 30–40 years. Healthcare providers must assess ASCVD risk forming the basis for prevention strategies tailored to individual risk profiles for optimal outcomes.

Risk communication is pivotal, empowering patients through shared decision-making and involving them in treatment choices. Open dialogues should consider patient characteristics, preferences, and potential risks, guiding them toward choices aligned with their values and health objectives. For those at high ASCVD risk, behavioral counseling is essential, promoting personal responsibility and guiding lifestyle changes that significantly lower risk.

The importance of healthy behavior such as physical activity, proper nutrition, and smoking cessation, where applicable, cannot be overstated. These lifestyle modifications are the basis for managing ASCVD risk but are often hampered by difficulties in adherence. Healthcare providers play a vital role in supporting patients to make healthier choices, such as avoiding stress, quitting smoking and engaging in regular physical activity.

Furthermore, personalized pharmacological interventions are crucial, requiring careful consideration of each patient’s unique clinical profile, including comorbidities and potential drug interactions. This personalization extends to managing, e.g., blood pressure, where comprehensive risk assessments inform medication choices. Another example is the assessment of the risks and benefits of antiplatelet and anticoagulant medications based on individual patient factors.

Emerging pharmacological options such as PCSK9 inhibitors and SGLT2 inhibitors represent a promising evolution in ASCVD prevention strategies. While not widely included in current CPGs on primary prevention, these agents demonstrate significant cardiovascular benefits in high-risk populations (53). Incorporating evidence on their cost-effectiveness, long-term safety, and specific clinical indications into guidelines is crucial for optimizing prevention strategies. Future updates should systematically address the evidence for these agents to guide clinical decision-making effectively.

### 4.1 Strengths

In our systematic guideline review, we implemented a comprehensive search strategy that included an exploration of relevant electronic literature databases, supplemented by a sensitive search approach, and a manual review of databases and websites specifically dedicated to guidelines. This thorough examination ensured that we included guidelines varying in scope and focus, allowing us to map out the contemporary “landscape” of primary prevention of ASCVD in primary care. To our knowledge, no other systematic guideline review has covered all three domains of primary ASCVD prevention for a primary care population.

To assess the quality of the included guidelines, we adopted a structured two-step process developed by Muth et al. (14, 15). Initially, we applied the mini-checklist (MiChe) (24, 25) to evaluate and rate the quality of the guidelines. This checklist provided specific criteria that helped establish a framework for evaluation.

Following the initial quality assessment, we carried out a consistency analysis using the qualitative data analysis software ATLAS.ti (22). This tool was instrumental in managing and analyzing the heterogeneous data sets, allowing us to navigate and interpret the complex information effectively. Additionally, it allowed us to conduct a sub-group analysis to delve deeper into specific categories of interest, further refining our understanding and assessment of the guidelines.

These methodologies were employed to provide a reliable and thorough synthesis of the available evidence and recommendations for the primary prevention of cardiovascular disease. By integrating these approaches, our findings can offer feedback to authors and users of the included guidelines as well as insightful guidance for future guideline development for clinical practice.

### 4.2 Limitations

Our guideline review, while comprehensive, faced certain limitations that are important to acknowledge. A primary limitation was due to the lack of a universally accepted classification system for grading the quality of evidence and determining the strength of recommendations. Different organizations use diverse systems such as GRADE and modified OCEBM (54, 55), which lead to variations in how recommendations were categorized and interpreted across different guidelines. This lack of standardization created inconsistencies in evidence grading and recommendation strength, complicating the comparison and synthesis of guideline data.

Moreover, interpreting recommendations across these different grading systems can be challenging, as similar terms or labels may carry different meanings depending on the grading system used. This variability can lead to potential misinterpretation and misapplication of the guidelines, affecting clinical decision-making processes. The intricate nature of clinical evidence, which includes a mix of randomized controlled trials, observational studies, and expert opinions, adds further complexity. Integrating these diverse evidence sources requires nuanced judgment that can introduce subjectivity and inconsistencies into guideline recommendations.

Additionally, the format and layout of published guidelines often do not support straightforward tracking of the evidence supporting individual recommendations. This issue was particularly evident during our consistency analysis, even among high-quality guidelines assessed using the MiChe checklist (24, 25). The challenges posed by varied grading systems and the complex nature of clinical evidence complicate the creation and application of consistent, easily interpretable clinical guidelines.

## 5 Conclusion

Our systematic guideline review provides a comprehensive overview of global strategies for the primary prevention of ASCVD based on up-to-date evidence. It has highlighted the diversity in the content and presentation of guidelines, and unexpectedly revealed a high degree of heterogeneity in recommendations, which poses challenges for developing and updating clinical practice guidelines. Few topics were found to be directly applicable or suitable for straightforward adaptation, indicating a need for significant refinement and localization of international recommendations to respective national contexts. At the same time, this highlights the importance of a transparent evidence-to-decision process, which lays open the considerations of different parties involved in phrasing the recommendations.

The strength of the recommendations within these guidelines often did not reliably indicate consistent topics, largely due to inconsistent interpretations that frequently stem from conflicting grades or levels of evidence. This issue is exacerbated by the varied classification systems used across CPGs, which complicates the analysis and can lead to inconsistent guideline applications, ultimately impacting the quality of patient care.

To address these challenges, there is a critical need for harmonization and standardization in the development of guidelines to enhance their comparability, usability, and consistency. Initiatives such as the Guidelines International Network (G-I-N) and the GRADE framework provide valuable models for achieving consistency and transparency in guideline development.

Our findings may support guideline developers in deciding about appropriate frameworks, delimiting areas of focus, and phrasing clinically relevant questions. While existing guidelines often lack clear links between the evidence levels and the studies cited, our data allows for identifying key publications on prioritized topics as a foundation or reference point in conducting independent evidence searches and evaluations. Alignment with the GRADE criteria subsequently helps to ensure that the process is transparent and that the recommendations for the primary prevention of ASCVD are sound and effectively tailored to meet the specific needs of the population.

This approach can lay a solid foundation for the development of reliable and effective ASCVD prevention guidelines, thereby improving cardiovascular health outcomes and promoting well-being across a wider population.

## Data Availability

The raw data supporting the conclusions of this article will be made available by the authors, without undue reservation.
