# Supplementary material for: Recommendations for the primary prevention of atherosclerotic cardiovascular disease in primary care: a systematic guideline review
Source: Front Med (Lausanne). 2025 Jan 21;11:1494234. doi: 10.3389/fmed.2024.1494234 (PMC11792287; doi:10.3389/fmed.2024.1494234)
Supplement: Supplementary file 1 [file Supplementary_file_1.docx]

**Identification of studies via other methods**

**Identification of CPGs via electronic databases**

Records identified from:

CPG databases (n = 1,484)

Scientific Societies (n = 533)

Others (n = 695)

Records removed *before screening*:

Duplicate records removed (n = 86)

Records identified from:

Databases (n = 2,411)

(MEDLINE n = 1,042, TRIP n = 1,290)

**Identification**

Records screened

(n = 2,325)

Records excluded

(n = 2,147)

Reports not retrieved

(n = 16)

Reports sought for retrieval

(n = 928)

Reports sought for retrieval

(n = 178)

Reports not retrieved

(n = 2)

**Screening**

Reports assessed for eligibility

(n = 912)

Reports excluded:

Wrong publication (n = 47)

Wrong population (n = 94)

Wrong intervention (n = 9)

Wrong topic (n = 341)

Duplicates (n = 415)

Reports assessed for eligibility

(n = 176)

Reports excluded:

Wrong publication (n = 53)

Wrong population (n = 5)

Wrong intervention (n = 5)

Wrong topic (n = 57)

Other (n= 2)

Duplicates (n = 34)

Reports included in review

(n = 20 electronic databases; n = 6 hand searching)

CPG of included studies

(n = 26)

**Included**

*From:*  Page MJ, McKenzie JE, Bossuyt PM, Boutron I, Hoffmann TC, Mulrow CD, et al. The PRISMA 2020 statement: an updated guideline for reporting systematic reviews. BMJ 2021;372:n71. doi: 10.1136/bmj.n71. For more information, visit: <http://www.prisma-statement.org/>
