# Supplementary material for: Recommendations for the primary prevention of atherosclerotic cardiovascular disease in primary care: a systematic guideline review
Source: Front Med (Lausanne). 2025 Jan 21;11:1494234. doi: 10.3389/fmed.2024.1494234 (PMC11792287; doi:10.3389/fmed.2024.1494234)
Supplement: Supplementary file 3 [file Table_2.docx]

## Table S2. Categories of consistency and inconsistency

| **Type** |  | **Definition** | **Further Action Plan for Guidance Development** |
| --- | --- | --- | --- |
| **Consistency** |  |  |  |
| (1) | **High consistency** | **Recommendations are consistent**in content, evidence level and grading, and **based on a large body of high-level evidence** (e.g., multiple primary or secondary studies of high internal and external validity) | Verification of cited sources with highest evidence level, and update searches, if necessary |
| (2) | **Moderate consistency** | **Recommendations are consistent**in content, evidence level and grading, and **based on a small body of high-level evidence** (e.g. a single or a few primary studies of high internal and external validity) | Verification of cited sources, further research on safety aspects in particular, and update searches |
| (3) | **Partial consistency** | **Recommendations are consistent** in content, evidence level and grading, and **based on** evidence from studies of **low-level evidence**(e.g. studies with design-related biases or where methodological flaws reduce internal or external validity) **or based on expert opinion** (where evidence is lacking) | Further research on evidence |
| (4) | **Low consistency** | **Recommendations are consistent** in content, but evidence levels and grading conflict | Verification of cited sources, and update searches |
| **Inconsistency** |  |  |  |
| (A) | **High inconsistency** | **Recommendations are completely inconsistent**, neither a mainstream trend nor even a common denominator can be identified | Further research on evidence |
| (B) | **Moderate inconsistency** | **Recommendations are**consistent in the majority of guidelines, but **differing** or even conflicting **recommendations are to be found in a minority** | Verification of cited sources to decide whether further research is necessary, and update-searches |
| (C) | **Low inconsistency** | **Recommendations** cover a **common topic or even have a common trend,** but put forward **different specific aspects or a combination of those** | Verification of cited sources to decide whether further research is necessary, and update-searches |

Modified from source: Muth C, Gensichen J, Beyer M, Hutchinson A, Gerlach FM. The Systematic Guideline Review: Method, rationale, and test on chronic heart failure. BMC Health Serv Res. 2009 Dec;9(1):74.
