# Supplementary material for: Recommendations for the primary prevention of atherosclerotic cardiovascular disease in primary care: a systematic guideline review
Source: Front Med (Lausanne). 2025 Jan 21;11:1494234. doi: 10.3389/fmed.2024.1494234 (PMC11792287; doi:10.3389/fmed.2024.1494234)
Supplement: Supplementary file 4 [file Table_3.docx]

## Table S3. Key characteristics of the included studies

| **Developer (Acronym)** | **Title** | **Country** | **Target audience** | **Target population** | **Outcomes** |
| --- | --- | --- | --- | --- | --- |
| Management of ASCVD risk factors in general | | | | | |
| ACC/AHA 2019 | ACC/AHA Guideline on the Primary Prevention of Cardiovascular Disease | USA | Healthcare professionals | Adults with and without ASCVD | Morbidity and mortality from ASCVD as well as heart failure and atrial fibrillation |
| Canadian Cardiovascular Harmonized (CCH) 2022 | Canadian Cardiovascular Harmonized National Guideline Endeavour (C-CHANGE) guideline for the prevention and management of cardiovascular disease in primary care: 2022 update | Canada | Primary care professionals | Adults with multimorbidity | Morbidity and mortality from ASCVD |
| Ministry of Health Malaysia (MoH MY) 2017 | Clinical Practice Guidelines on Primary & Secondary Prevention of Cardiovascular Disease 2017 | Malaysia | Healthcare professionals | Adults with and without ASCVD | Morbidity and mortality from ASCVD |
| NICE 2014, revised 2023 | Cardiovascular disease: risk assessment and reduction, including lipid modification | UK | Healthcare professionals | Adults 18 or older without ASCVD | Morbidity and mortality from ASCVD |
| SIGN 2017 | Risk estimation and the prevention of cardiovascular disease | UK | Healthcare professionals | Adults with and without ASCVD | Morbidity and mortality from ASCVD |
| USPSTF 2021a | Screening for Hypertension in Adults | USA | Primary care professionals | Adults 18 or older without ASCVD | Morbidity and mortality from ASCVD |
| Management of ASCVD risk factors in rheumatic disease | | | | | |
| European League Against Rheumatism (EULAR) Task Force 2016 | EULAR recommendations for cardiovascular disease risk management in patients with rheumatoid arthritis and other forms of inflammatory joint disorders: 2015/2016 update. | Europe | Rheumatologist | Adults with rheumatic disease | Morbidity and mortality from ASCVD |
| Management of ASCVD risk factors in psychological diseases | | | | | |
| BAP 2016 | BAP guidelines on the management of weight gain, metabolic disturbances and cardiovascular risk associated with psychosis and antipsychotic drug treatment. | UK | Healthcare professionals | Adults 18 or older without ASCVD with psychosis | Morbidity and mortality from ASCVD and metabolic disorders |
| Management of prediabetes / diabetes | | | | | |
| ADA/ESE 2019 | Primary Prevention of ASCVD and T2DM in Patients at Metabolic Risk: An Endocrine Society* Clinical Practice Guideline | USA | Healthcare professionals | Adults without ASCVD with metabolic syndrome | Morbidity and mortality from ASCVD and type 2 diabetes mellitus |
| SBD/SBC/SBEM 2017 | Brazilian guidelines on prevention of cardiovascular disease in patients with diabetes: a position statement from the Brazilian Diabetes Society (SBD), the Brazilian Cardiology Society (SBC) and the Brazilian Endocrinology and Metabolism Society (SBEM) | Brazil | Healthcare professionals | Adults without ASCVD with type 1 or type 2 diabetes | Non-clinical, subclinical and clinical ASCVD |
| USPSTF 2021c | Screening for Prediabetes and Type 2 Diabetes | USA | Primary care professionals | Adults 35- 70 without ASCVD with obesity | Morbidity and mortality from ASCVD |
| Management of dyslipidemia | | | | | |
| British Medical Journal (BMJ) Rapid Recommendations 2022 | PCSK9 inhibitors and ezetimibe for the reduction of cardiovascular events: a clinical practice guideline with risk-stratified recommendations | UK | Healthcare professionals | Adults 40 or older without ASCVD with dyslipidemia with high dose statins or intolerant | Major ASCVD events |
| USDVA/USDoD 2020 | Management of Dyslipidemia for Cardiovascular Disease Risk Reduction | USA | Healthcare professionals | Adults 40 or older without ASCVD | Morbidity and mortality from ASCVD |
| USPSTF 2022b | Statin Use for the Primary Prevention of Cardiovascular Disease in Adults | USA | Primary care professionals | Adults 40 or older without ASCVD with dyslipidemia | Morbidity and mortality from ASCVD and all-cause mortality |
| Management of weight loss | | | | | |
| USPSTF 2018b | Behavioral Weight Loss Interventions to Prevent Obesity-Related Morbidity and Mortality in Adults | USA | Primary care professionals | Adults 18 years or older | Obesity-Related Morbidity and Mortality |
| Promote healthy behaviors | | | | | |
| SEN 2021 | Recommendations of the Spanish Society of Neurology for the prevention of stroke. Interventions on lifestyle and air pollution. | Spain | Neurologists, public health authorities | Adults without stroke | Morbidity and mortality from stroke |
| SINU 2018 | Diet and primary prevention of stroke: Systematic review and dietary recommendations by the ad hoc Working Group of the Italian Society of Human Nutrition. | Italy | Healthcare professionals and all interested readers | Adults without stroke | Morbidity and mortality from stroke |
| USPSTF 2020 | Behavioral Counseling Interventions to Promote a Healthy Diet and Physical Activity for Cardiovascular Disease Prevention in Adults With Cardiovascular Risk Factors | USA | Primary care professionals | Adults 18 or older without ASCVD with ASCVD risk factors | Morbidity and mortality from ASCVD |
| USPSTF 2022e | Behavioral Counseling Interventions to Promote a Healthy Diet and Physical Activity for Cardiovascular Disease Prevention in Adults Without Cardiovascular Disease Risk Factors: US Preventive Services Task Force Recommendation Statement. | USA | Primary care professionals | Adults 18 or older without ASCVD | Morbidity and mortality from ASCVD |
| Benefits and harms of using non-traditional risk factors | | | | | |
| USPSTF 2018a | Screening for Cardiovascular Disease Risk With Electrocardiography | USA | Primary care professionals | Adults 18 or older without ASCVD | Morbidity and mortality from ASCVD |
| USPSTF 2018c | Risk Assessment for Cardiovascular Disease With Nontraditional Risk Factors | USA | Primary care professionals | Adults 18 or older without ASCVD | Morbidity and mortality from ASCVD |
| USPSTF 2021b | Screening for Asymptomatic Carotid Artery Stenosis | USA | Primary care professionals | Adults 18 or older without ASCVD | Morbidity and mortality from ASCVD |
| Benefits and harms of hormone replacement therapy | | | | | |
| SOGC 2021 | Guideline No. 422e: Menopause and Cardiovascular Disease. | Canada | Healthcare professionals | Perimenopausal and postmenopausal women | Morbidity and mortality from ASCVD and venous thromboembolism |
| USPSTF 2022c | Hormone Therapy for the Primary Prevention of Chronic Conditions in Postmenopausal Persons | USA | Primary care professionals | Perimenopausal and postmenopausal women | Chronic diseases prevention |
| Benefits and harms of aspirin | | | | | |
| USPSTF 2022d | Aspirin Use to Prevent Cardiovascular Disease: US Preventive Services Task Force Recommendation Statement | USA | Primary care professionals | Adults 40 or older without ASCVD | ASCVD events and mortality, and all-cause mortality |
| Benefits and harms of vitamin, mineral, and multivitamin supplementation | | | | | |
| USPSTF 2022a | Vitamin, Mineral, and Multivitamin Supplementation to Prevent Cardiovascular Disease and Cancer | USA | Primary care professionals | Adults 18 or older without ASCVD | ASCVD, cancer, and mortality |
