# Supplementary material for: Recommendations for the primary prevention of atherosclerotic cardiovascular disease in primary care: a systematic guideline review
Source: Front Med (Lausanne). 2025 Jan 21;11:1494234. doi: 10.3389/fmed.2024.1494234 (PMC11792287; doi:10.3389/fmed.2024.1494234)
Supplement: Supplementary file 5 [file Table_4.docx]

## Table S4. Quality appraisal of included guidelines

| ***MiChe items***  ***Guidelines*** | 1. Identification of key recommendations and comprehensibleness | 2. Specification of the guideline’s target audiences and scope | 3. Specification of the objectives and the target population | 4. Independence and potential conflicts of interests | 5. Systematic search for evidence and selection criteria | 6. Unambiguity of recommendations | 7.  Different treatment options according to potential benefits, side effects and risks | 8. Information on update procedures | Overall assessment^[[1]](#footnote-1)^ |
| --- | --- | --- | --- | --- | --- | --- | --- | --- | --- |
| ACC/AHA 2019 | Yes | Yes | Yes | Yes | To some extent | Yes | Yes | To some extent | 2 |
| ADA/ESE 2019 | Yes | Yes | Yes | Yes | Yes | Yes | Yes | To some extent | 1 |
| BAP 2016 | Yes | Yes | Yes | Yes | No | Yes | Yes | To some extent | 3 |
| SBD/SBC/SBEM 2017 | Yes | To some extent | Yes | Yes | To some extent | Yes | Yes | To some extent | 3 |
| BMJ Rapid Reviews 2022 | Yes | To some extent | Yes | Yes | Yes | Yes | Yes | Yes | 1 |
| CCH 2022 | Yes | Yes | Yes | Yes | No | To some extent | Yes | Yes | 3 |
| EULAR 2016 | Yes | To some extent | Yes | Yes | Yes | Yes | Yes | To some extent | 1 |
| SINU 2018 | Yes | Yes | Yes | Yes | Yes | Yes | Yes | To some extent | 1 |
| Ministry of Health Malaysia (MoH MY) 2017 | Yes | Yes | Yes | Yes | To some extent | Yes | Yes | Yes | 1 |
| NICE 2014, revised 2023 | Yes | Yes | Yes | Yes | Yes | Yes | Yes | Yes | 1 |
| SIGN 2017 | Yes | Yes | Yes | Yes | To some extent | Yes | Yes | Yes | 1 |
| SOGC 2021 | Yes | Yes | Yes | To some extent | No | Yes | Yes | To some extent | 4 |
| SEN 2021 | Yes | To some extent | Yes | To some extent | To some extent | Yes | Yes | To some extent | 4 |
| USDVA/ USDoD 2020 | Yes | Yes | Yes | To some extent | Yes | Yes | Yes | To some extent | 2 |
| USPTF 2018a | Yes | Yes | Yes | Yes | Yes | Yes | Yes | Yes | 1 |
| USPTF 2018b | Yes | Yes | Yes | Yes | Yes | Yes | Yes | Yes | 1 |
| USPTF 2018c | Yes | Yes | Yes | Yes | Yes | Yes | Yes | Yes | 1 |
| USPTF 2020 | Yes | Yes | Yes | Yes | Yes | Yes | Yes | Yes | 1 |
| USPTF 2021a | Yes | Yes | Yes | Yes | Yes | Yes | Yes | Yes | 1 |
| USPTF 2021b | Yes | Yes | Yes | Yes | Yes | Yes | Yes | Yes | 1 |
| USPTF 2021c | Yes | Yes | Yes | Yes | Yes | Yes | Yes | Yes | 1 |
| USPTF 2022a | Yes | Yes | Yes | Yes | Yes | Yes | Yes | Yes | 1 |
| USPTF 2022b | Yes | Yes | Yes | Yes | Yes | Yes | Yes | Yes | 1 |
| USPTF 2022c | Yes | Yes | Yes | Yes | Yes | Yes | Yes | Yes | 1 |
| USPTF 2022d | Yes | Yes | Yes | Yes | Yes | Yes | Yes | Yes | 1 |
| USPTF 2022e | Yes | Yes | Yes | Yes | Yes | Yes | Yes | Yes | 1 |

1. On a scale from 1 = “very good” to 7 = “very poor” [↑](#footnote-ref-1)
