# Supplementary material for: Recommendations for the primary prevention of atherosclerotic cardiovascular disease in primary care: a systematic guideline review
Source: Front Med (Lausanne). 2025 Jan 21;11:1494234. doi: 10.3389/fmed.2024.1494234 (PMC11792287; doi:10.3389/fmed.2024.1494234)
Supplement: Supplementary file 6 [file Table_5.docx]

## Table S5. Strength of recommendations and level of evidence: Comparison across guidelines

**Table S5.a. Strength of recommendation: Comparison across guidelines**

| **Strength of Recommendation**  **Guideline** | **Strong recommendation for** | **Weak recommendation for** | | **No recommendation** | **Weak recommendation against** | **Strong recommendation against** |
| --- | --- | --- | --- | --- | --- | --- |
| **ACC/AHA 2019** | Class I  Benefit >>> Risk | Class IIa (Moderate) Benefit >> Risk | Class IIb (Moderate)  Benefit ≥ Risk | Class III: No benefit (Moderate)  Benefit = Risk |  | Class III Harm (Strong)  Risk > Benefit |
| **ADA/ESE 2019** | Strong recommendation for | Conditional recommendation for | |  | Conditional recommendation against | Strong recommendation against |
| **BAP 2016** | A (based on cat. I evidence) | B (based on cat. II evidence | C/D (based on cat. III/ IV evidence) |  |  |  |
| **BMJ Rapid Reviews 2022** | Strong recommendation for | Weak recommendation for | |  | Weak recommendation against | Strong recommendation against |
| **CCH 2022** | 1A, 1B, 1C | 2A, 2B, 2C | C/D |  |  |  |
|  | A | B |  |  |  |  |
| **EULAR 2016** | A | B | C/D |  |  |  |
| **Ministry of Health Malaysia 2017** | I | IIa | IIb |  |  | III |
| **NICE 2014, revised 2023** | Offer/ advice or ask about | Consider | |  |  | 'do not offer', 'advise', or 'ask about' |
| **SBD/SBC/SBEM 2017** | Class I | Class IIa | Class IIb |  |  | Class III |
| **SEN 2021** | Class I | Class IIa | Class IIb |  |  | Class III |
| **SIGN 2017** | Strong recommendation for | Conditional recommendation for | |  |  | Strong recommendation against |
| **SINU 2018** | Strong recommendation for | Weak recommendation for | |  | Weak recommendation against | Strong recommendation against |
| **SOGC 2021** | Strong recommendation for | Weak recommendation for | |  | Weak recommendation against | Strong recommendation against |
| **USDVA_USDoD 2020** | Strong recommendation for | Weak recommendation for | |  | Weak recommendation against | Strong recommendation against |
| **USPSTF 2018-2022** | A | B | C | Inconclusive |  | D |

**Table 5b. Evidence levels: Comparison across guidelines**

| **Guideline** | **Strength of Evidence** | | | | | | | | |
| --- | --- | --- | --- | --- | --- | --- | --- | --- | --- |
| **ACC/AHA 2019** | A |  | B-R |  | B-NR | C-LD | C-LD |  | C-RO |
| **ADA/ESE 2019** | 1\|⌖⌖⌖⌖  2\|⌖⌖⌖⌖ | 1\|⌖⌖⌖⌖^*^  2\|⌖⌖⌖⌖^*^ | 1\|⌖⌖⌖0  2\|⌖⌖⌖0 | 1\|⌖⌖⌖0^**^  2\|⌖⌖⌖0^**^ | 1\|⌖⌖00^***^  2\|⌖⌖00^***^ | 1\|⌖000  2\|⌖000 |  | 1\|⌖000  2\|⌖000 |  |
| **BAP 2016** |  |  |  |  |  |  |  |  |  |
| **BMJ Rapid Reviews 2022** |  |  |  |  |  |  |  |  |  |
| **CCH 2022** | Strong / 1a / 1A / High | 1B / 1 | 2 / 1b / Moderate | 2 / 2a | 3 /2a / Low |  | 3 | 3 | D / Consensus / 4 |
| **EULAR 2016** |  |  |  |  |  |  |  |  |  |
| **Ministry of Health Malaysia 2017** | A | B | | | | | | C | |
| **NICE 2014, revised 2023** |  |  |  |  |  |  |  |  |  |
| **SBD/SBC/SBEM 2017** | A | B | | | | | | C | |
| **SEN 2021** | A |  | B | |  |  | C |  |  |
| **SIGN 2017** | A |  | B | |  |  | C |  |  |
| **SINU 2018** | 1++ | 2++ | 1+ | 2+ |  | 1- | 2- | 3 | 4 |
| **SOGC 2021** | High |  | Moderate |  | Low | Very low |  |  |  |
| **USDVA_USDoD 2020** |  |  |  |  |  |  |  |  |  |
| **USPSTF 2018-2022** | High |  | Moderate |  | Low | | | |  |

^*^ Includes very strong evidence from unbiased observational studies.

^**^ Includes strong evidence from unbiased observational studies.

^***^ Includes some evidence from unbiased observational studie
