# Supplementary material for: Recommendations for the primary prevention of atherosclerotic cardiovascular disease in primary care: a systematic guideline review
Source: Front Med (Lausanne). 2025 Jan 21;11:1494234. doi: 10.3389/fmed.2024.1494234 (PMC11792287; doi:10.3389/fmed.2024.1494234)
Supplement: Supplementary file 7 [file Table_6.docx]

Table S6. Clinical questions from international primary prevention of atherosclerotic cardiovascular disease guidelines.

**Table S6.1. Risk assessment and patient-provider interaction**

| **Clinical questions / Clusters (Topics)** | **Number of guidelines supporting** | **Consistency/ Inconsistency** |
| --- | --- | --- |
| 1. Attribution | | |
| - 1. Who should have ASCVD risk assessment? |  |  |
| - Aged-based risk assessment | 5 | 4 |
| - Condition-based risk assessment | 4 | C |
| - Global risk-based risk assessment | 1 | nr |
| - 1. What are the benefits and harms of screening for a single risk factor or condition? |  |  |
| - Screening for atherosclerotic plaques/ stenosis in carotid arteries by use of carotid ultrasound | 2 | A |
| - Screening for diabetes | 3 | B |
| - Screening for hyper-/ dyslipidemia | 2 | A |
| - Screening for hypertension | 1 | nr |
| - Screening for obesity | 4 | A |
| - Screening for smoking/ tobacco use | 2 | A |
| - Screening for arterial stiffness | 1 | nr |
| - Screening for presence of CAD by use of non-invasive Tests (ECG - Resting or Exercise-, stress tests, CCTA) | 4 | A |
| - 1. What are the benefits and harms of using systematic *versus* opportunistic ASCVD risk assessment strategy? | 3 | A |
| - 1. When should ASCVD risk be reassessed? | 2 | C |
| 1. Calculate definitive risk | | |
| - 1. Should specific groups generally be considered at high risk? | 5 | C |
| 1. Basic risk calculation | | |
| - 1. What are the benefits and harms of using a standardized risk assessment tool assess ASCVD risk? | 6 | C |
| - 1. What are the benefits and harms of considering additional, non-traditional risk factors to determine global ASCVD risk? |  |  |
| - Coronary artery calcium | 4 | A |
| - Ankle-brachial index | 2 | 3 |
| - High-sensitivity C-reactive protein (hsCRP) | 2 | 3 |
| - Psychosocial factors | 2 | B |
| - Body mass index | 1 | nr |
| - Existing medical conditions and treatments | 2 | nr |
| - 1. Which cholesterol measures should be used? | 5 | C |
| - 1. How comprehensively should the ASCVD risk be assessed? -follow assessment tool -comprehensive patient history | 1 1 | nr nr |
| 1. Advanced risk calculation | | |
| - 1. Should lifetime or 30-year ASCVD risk be considered to guide decisions about preventive strategies? | 1 | nr |
| - 1. What are the benefits and harms of considering additional, non-traditional risk factors to reclassify persons with intermediate or borderline risk to guide decisions about preventive strategies? |  |  |
| - General | 2 | 3 |
| - Ankle Brachial Index | 1 | nr |
| - Carotid Intima-Media Thickness Test | 1 | nr |
| - ECG | 1 | nr |
| - High-sensitivity C-reactive protein | 1 | nr |
| - Coronary artery calcium score | 3 | 2 |
| 1. Assessment threshold | | |
| - 1. Which risk thresholds are stated to recommend BP management interventions? |  |  |
| - Non-pharmacological intervention | 1 | nr |
| - Pharmacological intervention | 1 | nr |
| - Non-pharmacological and pharmacological intervention | 1 | nr |
| - 1. Which risk thresholds are stated to recommend antithrombotic interventions? |  |  |
| - Pharmacological intervention | 3 | C |
| - 1. Which risk thresholds are stated to recommend lipid management interventions? |  |  |
| - Pharmacological intervention   1. What is the general relationship of ASCVD risk and various treatment options? | 8 | C |
| - Non-pharmacological and pharmacological intervention | 2 | 3 |
| - Hypertension but low risk | 1 | nr |
| - Asymptomatic but high risk | 1 | nr |
| - Statin indication - sex *versus* cardiovascular risk | 1 | nr |
| 1. Patient-provider interaction – Decision making | | |
| - 1. Should a shared decision-making approach be used? |  |  |
| - Statin therapy | 3 | C |
| - Aspirin Therapy | 1 | nr |
| - General - Patients declining recommended treatment | 2  1 | C  nr |
| - 1. Should absolute risk be used as basis of decision-making? - Individualized, visualized, numerical information | 1 | nr |
| - 1. How can patients be encouraged to participate in reducing their ASCVD risk? - explore patient's knowledge and beliefs - individualized referral to behavioral counseling | 1 1 | nr  nr |
| 1. Patient-provider interaction - Risk communication | | |
| - 1. How to communicate risk? -good patient experience -wording   2. How to document the communication? -assessment results and decisions made -shared management plan | 1 1  1 1 | nr nr  nr nr |

ASCVD = atherosclerotic cardiovascular disease; CAD = coronary artery disease; CCTA = coronary computed tomography angiography; ECG = electrocardiogram; na = not applicable; nr = not ratable.

**Table S6.2. Non-pharmacological interventions**

| **Clinical question** | **Clusters (Topics)** | **Number of guidelines supporting** | **Consistency/ Inconsistency** |
| --- | --- | --- | --- |
| 1. Counselling | | | |
| - 1. Who should be the recipients of lifestyle advice and behavioral counselling? | |  |  |
| - Adults at increased ASCVD risk | | 1 | nr |
| - 1. What is the recommended approach for delivering lifestyle advice and behavioral counselling effectively? | |  |  |
| - Accounting for personal circumstances | | 2 | C |
| - Emphasize benefits and show opportunities | | 2 | C |
| - Content and mode of behavioral counselling | | 3 | C |
| - Competencies for counselling | | 1 | nr |
| - Team-based care | | 2 | C |
| 1. Physical Activity | | | |
| - 1. What key principles should be considered when providing advice on physical activity? | |  |  |
| - Reduction of sedentary behavior | | 2 | 4 |
| - Encouragement of activity | | 2 | 4 |
| - Exercise at maximum safe capacity | | 2 | C |
| - 1. What modes of physical activity are beneficial to cardiovascular health? | |  |  |
| - Any physical activity | | 2 | 3 |
| - Incorporation of walking | | 3 | 3 |
| - Muscle strengthening | | 1 | nr |
| - 1. What levels of physical activity are beneficial to cardiovascular health? | |  |  |
| - Time of moderate or vigorous activity per week | | 4 | 3 |
| - Supportive tools | | 1 | nr |
| 1. Nutrition | | | |
| - 1. What dietary patterns are considered beneficial for promoting health and well-being? | |  |  |
| - Reference to national guidelines | | 4 | C |
| - Components of a healthy diet | | 4 | 4 |
| - Plant versus animal-based foods | | 1 | nr |
| - Limited sugar intake | | 1 | nr |
| - Reduce saturated fats, replace by unsaturated fats | | 3 | C |
| - 1. What general rules or guidelines are applicable for maintaining a healthy and balanced nutrition? | |  |  |
| - Reduce consumption of processed foods (sodium, refined carbohydrates, saturated fats) | | 4 | C |
| - 1. What are the recommended serving sizes or amounts of specific types of food that should be consumed for a balanced diet? | |  |  |
| - Whole grain | | 2 | 4 |
| - Fruits and vegetables | | 3 | 4 |
| - Fish | | 3 | C |
| - Olive oil and nuts | | 3 | C |
| - Legumes | | 1 | nr |
| - Meat | | 1 | nr |
| - Dairy products | | 1 | nr |
| - Coffee | | 1 | nr |
| - Tea | | 1 | nr |
| - Dark chocolate | | 1 | nr |
| - Unsweetened beverages / water | | 2 | 4 |
| - 1. What are the recommended amounts (or percentages of energy intake) of different fatty acids? | |  |  |
| - Limits of fat intake | | 3 | C |
| - 1. Which modes of intake for unsaturated fatty acids (dietary, supplements, preparations) are advisable? | |  |  |
| - Fat supplementation | | 5 | 4 |
| - Icosapent ethyl in persons with elevated triglycerides | | 2 | 4 |
| - 1. What level of dietary sodium intake is considered harmful or potentially detrimental to health? | |  |  |
| - Limits of sodium intake | | 3 | 4 |
| - Reduction of sodium | | 1 | nr |
| - 1. Which other plant-based components and modes of intake are considered relevant or important for maintaining a healthy diet? | |  |  |
| - Fiber | | 2 | 4 |
| - Plant extracts | | 2 | C |
| - 1. Which vitamins or provitamins are considered relevant for maintaining good health, and what are the recommended modes of intake for these nutrients? | |  |  |
| - Niacin / vitamin B3 | | 3 | 1 |
| - Folate (B9)- or B-vitamin | | 3 | B |
| - Vitamin C | | 1 | nr |
| - Antioxidant vitamins (B2, C, E) | | 2 | 4 |
| - Vitamin E or beta carotene (provitamin A) | | 1 | nr |
| - Vitamin D | | 1 | nr |
| - Multivitamin supplements | | 1 | nr |
| - Calcium | | 1 | nr |
| - Magnesium | | 1 | nr |
| - Potassium | | 1 | nr |
| - 1. What are the potential advantages and disadvantages or risks associated with the use of nutrient supplementation? | |  |  |
| - Single- or paired-nutrient supplements | | 1 | nr |
| 1. Nicotine use (tobacco smoke) | | | |
| - 1. How is nicotine use addressed or managed in the primary prevention of ASCVD? | |  |  |
| - Advice to stop smoking | | 6 | 4 |
| - Referral to smoking cessation services | | 5 | 4 |
| - Smoking cessation in persons with mental illness | | 1 | nr |
| - Pharmacotherapy for smoking cessation | | 2 | B |
| - e-cigarettes and shisha | | 1 | nr |
| - Prioritization of smoking cessation interventions | | 1 | nr |
| - 1. What is the rating or assessment of tobacco smoke in terms of its association with the risk of ASCVD? | |  |  |
| - Avoid passive smoking | | 4 | 4 |
| 1. Alcohol use | | | |
| - 1. How is alcohol consumption addressed or managed as part of the primary prevention strategy for ASCVD? | |  |  |
| - Screening for excess alcohol use | | 1 | nr |
| - Interventions regarding alcohol | | 2 | C |
| - Limits of alcohol intake | | 4 | B |
| - Assessment in people with schizophrenia | | 1 | nr |
| 1. Other measures | | | |
| - 1. What specific measures or interventions are implemented in relation to mental health? | |  |  |
| - Avoid chronic stress | | 1 | nr |
| - Mind-body-practices | | 1 | nr |
| - Therapy in case of anxiety or depression | | 2 | B |
| - Referral in case of complex psychological problems | | 2 | B |
| - 1. What are some alternative or additional treatment measures that are commonly considered or suggested? | |  |  |
| - Traditional or alternative medicine | | 1 | C |
| 1. Risk reduction various conditions / Diabetes | | | |
| - 1. What are the effective approaches or strategies for managing ASVCD risk? | |  |  |
| - Components of lifestyle change | | 1 | nr |
| - Lifestyle changes alone in persons at moderate risk | | 2 | 4 |
| - Assessment and management in special populations | | 3 | C |
| - 1. What are the effective approaches or strategies for managing cardiovascular risk in individuals with (pre-)diabetes? | |  |  |
| - Lifestyle modification in relation to medication | | 3 | C |
| - Lifestyle modification for persons at high risk of diabetes | | 3 | C |
| 1. Hypertension | | | |
| - 1. What are the effective approaches or strategies for managing cardiovascular risk in individuals with hypertension? | |  |  |
| - Lifestyle modification components | | 1 | nr |
| - Reduced salt intake | | 2 | 4 |
| - Relation of lifestyle changes and medication | | 2 | B |
| - Persons with persistent high blood pressure | | 1 | nr |
| - Blood pressure target | | 2 | B |
| - BP target in case of chronic kidney disease | | 1 | nr |
| 1. Dyslipidemia | | | |
| - 1. What are the effective approaches or strategies for managing cardiovascular risk in individuals with dyslipidemia? | |  |  |
| - Lifestyle modification in relation to medication | | 2 | C |
| - Target setting and monitoring | | 2 | A |
| - Counselling for adherence to medication | | 1 | nr |
| 1. Body weight | | | |
| - 1. What are the effective approaches or strategies for managing cardiovascular risk in individuals with excess body weight? | |  |  |
| - Weight control to improve risk profile | | 3 | 3 |
| - Weight reduction targets | | 2 | B |
| - Weight control strategies | | 2 | C |
| - Physical activity dosage for weight loss | | 2 | C |
| - Comprehensive interventions | | 4 | 4 |
| - 1. What additional therapeutic measures can be considered in managing cardiovascular risk in individuals with excess body weight? | |  |  |
| - Pharmacotherapy | | 1 | nr |
| - Bariatric surgery | | 2 | B |

ASCVD = atherosclerotic cardiovascular disease; BP = blood pressure; na = not applicable; nr = not ratable

**Table S6.3. Pharmacological interventions**

| **Clinical question** | **Clusters (Topics)** | **Number of guidelines supporting** | **Consistency/ Inconsistency** |
| --- | --- | --- | --- |
| 1. Statins | | | |
| - 1. Who should be treated with statins as blood cholesterol-lowering medication for the primary prevention of ASCVD? | |  |  |
| - General recommendation for using medication | | 3 | nr |
| - General recommendation for using statins | | 2 | C |
| - Patients with Familial hypercholesterolemia | | 1 | nr |
| - Elderly patients | | 2 | A |
| - Patients at borderline ASCVD risk | | 1 | nr |
| - Patients at intermediate ASCVD risk | | 3 | C |
| - Patients with high & very high ASCVD risk | | 2 | C |
| - Patients with CKD | | 2 | C |
| - Patients with Diabetes | | 1 | nr |
| - Patients with Diabetes & middle aged | | 1 | nr |
| - Patients with Diabetes & very high risk of ASCVR | | 1 | nr |
| - Patients with Diabetes & mixed dyslipidemia | | 3 | C |
| - Drug combination - Contraindication | | 3 | C |
| - 1. What are the recommended drug dosage and drug of choice when treating patients with statins as blood cholesterol-lowering medication for the primary prevention of ASCVD? | |  |  |
| - Dosage - General recommendation | | 2 | A |
| - Dosage - Middle-aged patients | | 1 | nr |
| - Dosage - Patients with high ASCVD risk & middle-aged | | 2 | A |
| - Dosage - Patients with high ASCVD risk | | 1 | nr |
| - Dosage - Patients with moderate ASCVD risk | | 1 | nr |
| - Dosage - Patients with moderate ASCVD risk & middle-aged | | 2 | C |
| - Dosage - Patients with diabetes & very high risk for ASCVD | | 2 | C |
| - Dosage - Patients with CKD | | 1 | nr |
| - Drug of choice - Patients with high risk for ASCVD | | 2 | 1 |
| - Drug of choice - Patients with diabetes | | 1 | nr |
| - Drug of choice - Contraindication | | 1 | nr |
| - Drug of choice - Elderly | | 1 | nr |
| - 1. What is the recommended target when treating patients with statins as blood cholesterol-lowering medication for the primary prevention of ASCVD? | |  |  |
| - General recommendation | | 2 | C |
| - Patients with diabetes & very high ASCVD risk | | 1 | nr |
| - Patients with diabetes & high ASCVD risk | | 2 | C |
| - Middle-aged patients | | 1 | nr |
| - 1. What is the recommended follow-up when treating patients with statins as blood cholesterol-lowering medication for the primary prevention of ASCVD? | |  |  |
| - Follow-up - Lipid profile review | | 3 | B |
| - Follow-up - Improving adherence | | 2 | C |
| - Follow-up - Improving adherence - adding medication | | 1 | nr |
| - Follow-up - Change of doses | | 1 | nr |
| - Follow-up - Interactions | | 1 | nr |
| - Follow-up - Monitoring | | 3 | C |
| - Follow-up - Adverse events | | 3 | C |
| 1. Non-statins - Fibrate | | | |
| - 1. What are the benefits and harms of using other blood lipid-lowering medication (i. e. fibrates) when used for the primary prevention of ASCVD? | |  |  |
| - Fibrates alone for primary preventing in general including people as well with CKD & diabetes | | 2 | 1 |
| - Combination of statins and fibrates | | 3 | 4 |
| - Hypertriglyceridemia | | 3 | C |
| 1. Non-statins - Nicotinic Acid | | | |
| - 1. What are the benefits and harms of using other blood lipid-lowering medication (i.e., nicotinic acid) when used for the primary prevention of ASCVD? - Nicotinic acid alone for primary prevention in general including as well people with CKD & diabetes - Combination of statins and nicotinic acid | | 2  1 | 4  nr |
| 1. Non-statins – Ezetimibe | | | |
| - 1. What are the benefits and harms of using other blood lipid-lowering medication (i. e. ezetimibes) when used for the primary prevention of ASCVD? | |  |  |
| - Primary hypercholesterolemia | | 2 | 4 |
| - High & very high risk for ASCVR | | 2 | C |
| - Diabetes & very high risk for ASCVR | | 1 | nr |
| - Moderate risk for ASCVR | | 1 | nr |
| - General recommendation with or without statins | | 1 | nr |
| 1. Non-statins – PCSK9 inhibitors | | | |
| - 1. What are the benefits and harms of using other blood lipid-lowering medication (i. e. PCSK9 inhibitors) when used for the primary prevention of ASCVD? | |  |  |
| - High & very high risk for ASCVR | | 3 | C |
| - Moderate risk for ASCVR | | 1 | nr |
| - General recommendation | | 1 | nr |
| 1. Non-statins – Bile acid sequestrants | | | |
| - 1. What are the benefits and harms of using other blood lipid-lowering medication when used for the primary prevention of ASCVD? | |  |  |
| - Bile acid sequestrants for the PP of ASCVD in general, for people with CKD and for people with type 2 diabetes - Bile acid sequestrants for the PP of ASCVD in general in combination with statins | | 1  1 | nr  nr |
| 1. Blood pressure | | | |
| - 1. Who should be treated with BP-lowering medication for the primary prevention of ASCVD? | |  |  |
| - Use for patients with high ASCVD risk | | 4 | C |
| - Use for patients with ASCVD risk < 10% | | 3 | C |
| - Use for patients with diabetes - Use in patients with rheumatic diseases | | 2  1 | C  nr |
| - 1. What are the recommended drug dosage and drug of choice when treating patients with BP-lowering medication for the primary prevention of ASCVD? | |  |  |
| - Drug of choice - monotherapy | | 2 | C |
| - Dosages - combinations | | 1 | nr |
| - Drug of choice - combinations | | 2 | C |
| - Drug of choice - diabetes & CKD | | 2 | C |
| - Drug of choice - contraindication | | 1 | nr |
| - Drug of choice - adverse events | | 1 | nr |
| - 1. What is the recommended target when treating patients with BP-lowering medication for the primary prevention of ASCVD? | |  |  |
| - General recommendation about BP targets | | 3 | C |
| - Target for patients with hypertension & low risk of ASCVD | | 2 | C |
| - Target for patients with hypertension & high risk of ASCVD | | 2 | C |
| - Target for patients with hypertension & diabetes | | 3 | 4 |
| - Target for patients with hypertension & diabetes - elderly population | | 1 | nr |
| - Target for patients with stage III hypertension & diabetes | | 1 | nr |
| - Target for patients with hypertension & diabetes - albuminuria | | 1 | nr |
| 1. ASA (aspirin) | | | |
| - 1. Does regular aspirin use in patients without known ASCVD reduce ASCVD and CRC incidence and mortality, or all-cause mortality? | |  |  |
| - Use in patients without known ASCVD | | 3 | 1 |
| - Use in patients with high ASCVD risk and middle-aged | | 3 | 4 |
| - Use for the elderly | | 2 | 2 |
| - Use in patients with diabetes | | 3 | 4 |
| - Use in patients with diabetes & high ASCVD risk, elderly, and low risk of bleeding | | 2 | 4 |
| - Use in patients with hypertension | | 1 | nr |
| - Use if patients have an increased risk of bleeding | | 1 | nr |
| - Summary of recommendations | | 1 | nr |
| - 1. Does the effect vary by dose or duration of aspirin use? | |  |  |
| - Use in patients with high ASCVD risk and middle-aged | | 2 | 4 |
| - Use for the elderly | | 2 | 2 |
| - Use in patients with increased risk of bleeding | | 1 | nr |
| - Summary of recommendations | | 1 | nr |
| - 1. Does regular antiplatelet treatment use in patients without known ASCVD reduce ASCVD and CRC incidence and mortality, or all-cause mortality? | |  |  |
| - Diabetes | | 1 | nr |
| 1. Diabetes | | | |
| - 1. What are the benefits and harms of pharmacotherapy for the treatment of diabetes when used for the primary prevention of ASCVD? | |  |  |
| - General recommendation | | 1 | nr |
| - 1. What is the recommended dosage and drug of choice for pharmacotherapy for the treatment of diabetes for the primary prevention of ASCVD? | |  |  |
| - Drug of choice - Monotherapy - Metformin | | 3 | C |
| - Drug of choice - Monotherapy - Sulfonylureas | | 1 | nr |
| - Drug of choice - Combination - Second drug | | 3 | C |
| - Drug of choice - Combination - Third drug | | 1 | nr |
| - Drug of choice - Monotherapy - Metformin intolerance | | 1 | nr |
| - Drug of choice - Insulin | | 2 | 4 |
| - Dosage - Acute illness | | 1 | nr |
| - Drug of choice - Monotherapy - Metformin - Prediabetes | | 2 | C |
| - Drug of choice - Monotherapy - Other - Prediabetes | | 1 | nr |
| - Drug of choice - Monotherapy - Other - Renal impairment | | 1 | nr |
| - 1. What is the recommended target of pharmacotherapy for the treatment of diabetes for the primary prevention of ASCVD? | |  |  |
| - General recommendation | | 2 | C |
| - Patients with CKD | | 1 | nr |
| 1. Overweight - Obesity | | | |
| - 1. What are the benefits and harms of pharmacotherapy for weight loss control when used for the primary prevention of ASCVD? | |  |  |
| - General recommendation for pharmacotherapy for weight loss | | 1 | nr |
| - Availability of anti-obesity drugs | | 1 | nr |
| - Orlistat for patients with psychosis and antipsychotic drug treatment - efficacy for weight loss | | 1 | nr |
| - Orlistat for patients with psychosis and antipsychotic drug treatment - long term use | | 1 | nr |
| - Orlistat for patients with psychosis and antipsychotic drug treatment - adherence | | 1 | nr |
| - Topiramate for patients with psychosis and antipsychotic drug treatment - efficacy for weight loss | | 1 | nr |
| - Metformin for patients with psychosis and antipsychotic drug treatment - efficacy for weight loss - Metformin for patients with psychosis and antipsychotic drug treatment – adverse events | | 1  1 | nr  nr |
| - Reboxetine for patients with psychosis and antipsychotic drug treatment - efficacy for weight loss | | 1 | nr |
| - Glucagon-like peptide-1 (GLP-1) for patients with psychosis and antipsychotic drug treatment - efficacy for weight loss | | 1 | nr |
| - Amantadine, melatonin and zonisamide for patients with psychosis and antipsychotic drug treatment - efficacy for weight loss | | 1 | nr |
| - Atomoxetine, dextroamphetamine, famotidine, fluoxetine, fluvoxamine and nizatidine for patients with psychosis and antipsychotic drug treatment - efficacy for weight loss | | 1 | nr |
| - Atomoxetine, dextroamphetamine, famotidine, fluoxetine, fluvoxamine and nizatidine - efficacy for weight loss | | 1 | nr |
| 1. Smoking | | | |
| - 1. What are the benefits and harms of pharmacotherapy for smoking cessation when used for the primary prevention of ASCVD? | |  |  |
| - Pharmacotherapy for smoking cessation in patients unable or unwilling to accept a referral | | 1 | nr |
| - Combining counselling with pharmacotherapy for patients that smoke- efficacy for smoking cessation - Varenicline or combination nicotine replacement therapy for patients that smoke- efficacy for smoking cessation - Bupropion and single nicotine replacement therapy for patients that smoke- efficacy for smoking cessation | | 2  1  1 | 4  nr  nr |
| - Nicotine replacement therapy for patients with psychosis and antipsychotic drug treatment that smoke - efficacy for smoking cessation | | 1 | nr |
| - Bupropion for patients with psychosis and antipsychotic drug treatment that smoke - efficacy for smoking cessation | | 1 | nr |
| - Varenicline for patients with psychosis and antipsychotic drug treatment that smoke - efficacy for smoking cessation | | 1 | nr |
| 1. Hormone replacement therapy | | | |
| - 1. What are the benefits and harms of menopausal hormone replacement therapy (HRT) when used for the primary prevention of ASCVD? | |  |  |
| - HRT for postmenopausal women to primary prevent ASCVD | | 3 | 4 |
| - If prescribed how to minimize risk of ASCVD | | 2 | C |
| - If prescribed how to avoid adverse events | | 1 | C |
| 1. Other diseases / health conditions | | | |
| - 1. What are the benefits and harms of the pharmacological treatment of other diseases? | |  |  |
| - Rheumatic diseases -NSAIDs | | 1 | nr |
| - Rheumatic diseases - cortocosteroids | | 1 | nr |
| - General recommendation | | 1 | nr |
| - Depression / anxiety | | 1 | nr |
| - Erectile dysfunction | | 1 | nr |
| - Testosterone replacement therapy | | 1 | nr |
| - Hormones anti-aging | | 1 | nr |
| - Alcohol dependence | | 1 | nr |
| - Persons with schizophrenia - bariatric surgery as alternative to medication | | 1 | nr |

ASA=Acetyl salicylic acid; ASCVD = atherosclerotic cardiovascular disease; ASCVR = atherosclerotic cardiovascular risk; BP = blood pressure; CKD = kidney disease; na = not applicable; nr = not ratable.

**Reviewers’ notes:**

“Authors did not specify active principle and dose.

^&^ Authors showed no changes in cardiovascular mortality with aspirin use but there were significant and clinically relevant changes in cardiovascular events favoring use of aspirin; however, gastrointestinal bleeding was increased by 46%.

$ Authors found a modest relative risk but the number needed to treat was high. Authors did not make any clear recommendations based on these results. We would suggest "weak recommendation against."
