# Supplementary material for: Recommendations for the primary prevention of atherosclerotic cardiovascular disease in primary care: a systematic guideline review
Source: Front Med (Lausanne). 2025 Jan 21;11:1494234. doi: 10.3389/fmed.2024.1494234 (PMC11792287; doi:10.3389/fmed.2024.1494234)
Supplement: Supplementary file 8 [file Table_7.docx]

Table S7. Demographic characteristics and health conditions addressed across the included guidelines

| **Subpopulations** | **Number of guidelines addressing the subpopulation** | **Number of recommendations addressing the subpopulation** |
| --- | --- | --- |
| 1. Demographic characteristics | | |
| - 1. Age | 10 | 17 |
| - 1. Sex | 6 | 24 |
| - 1. Gender | 0 | 0 |
| - 1. Ethnicity | 4 | 4 |
| 1. Health conditions | | |
| - 1. Diabetes | 9 | 95 |
| - 1. Other conditions | 10 | 86 |
| - 1. Multimorbidity | 2 | 7 |
| - 1. Polypharmacy | 8 | 42 |
